# Life-course patterning of MLTC clusters and their patient-centred burden in depression: a population-based study using real-world data

**DOI:** 10.1136/bmjment-2026-302705

**Published:** 2026-07-21

**Authors:** Alex Dregan, Thomas Canning, Marc Delord, Mozhdeh Shiranirad, Emilia Holland, Jakub Dylag, Michael Boniface, Mariam Molokhia, David Armstrong, Matthew Hotopf, Robin Poole, Frances S Mair, Nisreen A Alwan, Simon DS Fraser

**Affiliations:** 1 Department of Psychological Medicine, King’s College London Institute of Psychiatry Psychology & Neuroscience, London, England, UK; 2 Wolfson Sensory, Pain and Regeneration Centre, King’s College London Institute of Psychiatry Psychology & Neuroscience, London, England, UK; 3 King’s College London Department of Population Health Sciences, London, England, UK; 4 University of Southampton School of Mathematical Sciences, Southampton, England, UK; 5 University of Southampton School of Primary Care Population Sciences and Medical Education, Southampton, England, UK; 6 School of Electronics and Computer Science, University of Southampton, Southampton, UK; 7 Southampton City Council, Southampton, England, UK; 8 General Practice and Primary Care, University of Glasgow School of Health and Wellbeing, Glasgow, Scotland, UK; 9 University Hospital Southampton NHS Foundation Trust, Southampton, England, UK; 10 NIHR Applied Research Collaboration Wessex, Southampton, UK

## Abstract

**Background:**

Understanding how multiple long-term conditions (MLTCs) develop and cluster across the life-course remains a key public health priority. Evidence on how these patterns relate to patient-centred outcomes among patients with mental health disorders remains limited.

**Objective:**

To evaluate patient-centred outcomes in patients with a history of depression and MLTC.

**Methods:**

A retrospective matched cohort study design was implemented in the Clinical Practice Research Datalink, a UK primary care database. Patients aged ≥10 years with a first recorded diagnosis of depression between 1 January 2003 and 31 December 2023 were matched (1:1) to a comparator without a depression history on age, sex and practice. MLTC was defined using 256 long-term conditions. Clusters of MLTC were derived for each life decade via multiple correspondence analysis and k-means clustering. Patient-centred burden was assessed across eight patient-prioritised ‘work themes’: symptoms, emotions, investigation and monitoring, health service and administration, learning and adapting, medication, finance and accumulation and complexity. Poisson and logistic regression models assessed associations between decade-specific MLTC clusters and burden indicators.

**Findings:**

Among 1 962 757 patients with incident depression, 1 563 065 (78%) presented with MLTC over a mean follow-up of 14 years (SD=6), compared with 987 138 (50%) of comparators (mean follow-up=10, SD=7). Patients with depression history had a higher median number of LTCs (5 (IQR 3–9) vs 4 (3–8)). Clusters of MLTCs varied by life decade: atopic triad and liver clusters predominated in early life, metabolic and cardiovascular (CVD) clusters in midlife and musculoskeletal (MSK) and respiratory in later life. The burden patterning varied between cases and their comparators, who showed lower overall burden and distinct early-life patterns. The strongest prognostic cluster for increased burden in patients with depression changed across life. In early life, the metabolic cluster had the highest risk (incidence rate ratio=1.02 (95% CI 1.01 to 1.03)), followed by respiratory and CVD during midlife and MSK/functional in later life.

**Conclusions:**

MLTC in patients with depression is common, shows dynamic temporal patterning and leads to high patient-centred burden from adolescence onwards.

**Clinical implications:**

The study identified specific burdensome clusters of MLTC at each life decade which could inform MLTC prevention for patients with depression.

**WHAT IS ALREADY KNOWN ON THIS TOPIC**

- There is a growing evidence base examining how multiple long-term conditions (MLTCs) cluster across the life course.
- Depression has been related to increased MLTC and healthcare usage.
- There is growing interest in patient-centred ‘burden’ or workload indicators and how these relate to MLTC.

**WHAT THIS STUDY ADDS**

- This study extends existing evidence by tying an inclusive set of MLTCs to comprehensive and evaluated patient-centred burden indicators using primary care data.
- Depression was found to be associated with early and dynamic multimorbidity that confers substantial, stage-specific burden.
- Findings suggest that specific clusters of MLTCs were associated with highest overall patient-centred burden and that these varied by decade of life and by depression.

**HOW THIS STUDY MIGHT AFFECT RESEARCH, PRACTICE OR POLICY**

- This study indicates that patient-centred burden indicators can be evaluated in primary care data sets.
- It highlights the need for decade-tailored, integrated mental-physical prevention and care strategies.

## Background

 The growing number of people living with multiple long-term conditions (MLTCs) poses a major challenge for healthcare systems across the world.^1^ People with mental disorders are particularly vulnerable to early-onset complex patterns of MLTC,^2^ often experiencing a higher burden or ‘workload’ of disease, increased functional impairment and poorer clinical outcomes.^3–6^ Several studies have shown that the co-existence of physical and mental conditions contributes substantially to avoidable health service use and mortality.^7 8^ Yet, current knowledge of how clusters of MLTCs impact on patient-centred indicators of burden at specific life course is lacking, particularly among people who live with depression. Such insight is critical to the development of effective and timely preventative strategies to address the early onset of functional deterioration in people living with depression and complex MLTC.

The temporal patterning of MLTC burden is an important consideration for public health policy that has not been thoroughly explored in people with depression. Most studies model the consequences of MLTCs for patients as a static^9^ rather than a changing construct affected by multiple personal, sociodemographic and clinical factors as people transition across subsequent life stages.^10 11^ Current knowledge of when high-burden clusters of MLTC emerge and how their burden may evolve across specific life decades is thus incomplete. Population-based cohorts are often restricted to a small number of LTCs,^12^ predominantly those considered burdensome for the care system.^13^ This arbitrary selection criterion may inadvertently exclude less frequent LTCs or LTCs associated with specific social characteristics^14 15^ that may nevertheless place people at high levels of personal burden.^16^

## Objective

Leveraging longitudinal real-world primary care data, this study assessed the dynamic patterning of MLTC and patient-centred burden in people with a history of depression and their comparators without depression over the life course. First, we aimed to identify specific clusters of MLTCs at different life decades and define these with respect to a set of prespecified burden indicators for patients with depression and matched comparators. Second, we aimed to uncover MLTC clusters predictive of high overall and specific patient-centred burden indicators at each life decade.

## Methods

### Data and study participants

The present study used a retrospective matched cohort design using data from the Clinical Practice Research Datalink (CPRD) Aurum, between 1 January 2003 and 31 December 2023. CPRD Aurum, one of the largest electronic primary care databases, includes routinely collected medical records from around 20% of general practices in England covering ~17 million active patients (~52 million historical patients) and a few historical practices from Northern Ireland. The population is broadly representative of the UK primary care population in terms of age, gender and ethnicity.^17^ The CPRD collects detailed information, including diagnoses, symptoms, laboratory tests, prescriptions, referrals and service use. Although not routinely, it also collects non-clinical information (eg, health behaviours, employment and benefits).

### Study population

We included all patients aged 10 years and over, with a first recorded episode of depression between 01 January 2003 and 31 December 2023 and who were actively registered to a practice. Follow-up was restricted to codes issued when reporting practices had acceptable data quality, as per CPRD internal data completeness and validity flags. Follow-up ended at the earliest of death, de-registration and study end. The age inclusion criteria enabled us to estimate the burden of depression across the life course (before and after a depression event) from early adolescence to late adult years. We chose age 10 years to align with early depression onset (especially as age 16 years or 18 years would miss some depression onset) and to align with the age groupings used in this analysis. The index date was set as the date of first depression code. Each case was matched without replacement on a ratio of 1:1 to a comparator without depression or anxiety during the follow-up period, based on age at index date (within a 2-year window), sex and general practice to compare outcomes and burden between two groups. Each comparator was allocated the index date of their respective matched case to ensure temporal comparability (ie, ensure both cases and matched comparators have the same follow-up and exposure assessment period). This matching ensured comparability between case and comparator. A flow chart detailing cohort assembly is provided in online supplemental figure S1.

### Study variables

#### Long-term conditions and MLTC

A total of 256 long-term conditions (LTC; online supplemental material, table S1) were included to define MLTC, including mental, physical and infectious conditions. We excluded LTCs related to pain (eg, chest pain, generalised pain) and headache because these conditions are directly represented within the pre-specified burden indicators, and excluded anxiety due to its frequent co-occurrence and diagnostic overlap with depression. Our list of conditions includes both chronic and acute presentations (eg, appendicitis, gingivitis) to reflect real-world clinical practice. Acute conditions were retained as, for some patients, they may evolve into chronic disease or act as precipitating events for longer-term conditions. Some conditions may partially overlap (eg, angina, coronary artery disease); however, we included all to ensure comprehensive capture of the full spectrum of MLTC. To minimise double-counting bias we fully excluded medical codes that may be relevant to two or more related conditions (eg, liver disorders and cirrhosis). LTCs were defined using established MLTC codelists (OpenSAFELY, CALIBER, HDR UK and the CPRD MLTC repository), supplemented with updated Systematized Nomenclature of Medicine (SNOMED) codes identified via the CPRD browser and independently validated by at least two team members. All LTCs were coded as binary variables (0 or 1) for each decade for each patient, based on the presence of an appropriate code in that decade, irrespective of its timing related to the depression index date. Each SNOMED code was excluded from other codes to ensure no double counting of conditions.

### Depression

Depression was identified using validated SNOMED CT and Read (V.2) medical codes indicative of depressive disorders (including major depressive disorder, mild, moderate or severe depression). The selected codes were cross-checked with published code lists and validated by clinical experts on the team.

### Indicators of burden for MLTC

We used multiple and diverse indicators to define burden of MLTC within our study population. The selection and definition of these indicators have been detailed in a recent publication.^3^ Through a multistage process including both experienced researchers using CPRD and at least two clinical reviewers with experience of general practice, we generated a list of 56 indicators using SNOMED CT and Read medical codes and classified these into eight prespecified overarching ‘work themes’ to encapsulate different aspects of a patient’s life and care that are affected by living with MLTC. These themes were informed by the findings of a qualitative evidence synthesis of 46 studies^6^ among people living with MLTC. Full detail for the eight work themes is available in the online supplemental methods.

For example, the ‘accumulation’ work theme includes information on aspects related to care coordination (mental healthcare coordination plan), self-help (received relevant self-help guidance) and drug interactions (multiple drug side effects). The ‘emotions’ theme included a range of feelings such as fear, loneliness, self-esteem, suicidal thoughts, etc. The classification of specific codes into the eight work themes was informed by consultation with patients with lived experience and clinical guidance from a panel of clinicians with experience of coding in primary care. Each theme was defined as a binary variable, with (1) if a code for the theme was present in that decade and (0) if not. In addition, we created an *overall burden indicator* at each life decade to reflect the total functional and health-related impact of the burden indicators. The measure of burden was defined as the cumulative score of the eight specific burden indicators (range: 0 (none)–8 (all indicators)). The burden indicators were not restricted to those recorded after LTCs, as this would have substantially reduced the sample size. Moreover, the temporal sequencing of LTCs and burden indicators in primary care records is often complex, with both potentially recorded in varying order or concurrently. A full codelist is available online on the study repository (https://git.soton.ac.uk/meldb/).

### Covariates

Several available sociodemographic variables known to affect both the risk of MLTC and related outcomes were included as covariates. These comprised age, sex, ethnicity and region of registered general practitioner practice. Age was used to categorise participants into non-overlapping life decades (eg, 10–19 years, 20–29 years, …, 80–89 years and 90+ years). Ethnicity was derived from medical codes and grouped into broad categories: white, black, Asian, mixed and other. Region was defined according to the government office region of patient’s primary care practice (ie, North East, North West, Yorkshire and the Humber, East Midlands, West Midlands, East Anglia, South East, South West, London and Northern Ireland). As missingness in CPRD is generally not at random but reflects historical variation in recording practices, and because overall missingness was low, we conducted a complete case analysis.

### Statistical analysis

Descriptive statistics (frequencies, percentages, means) were used to compare baseline characteristics between patients with a history of depression and their matched comparator group. Mean follow-up years was calculated after study start (the latest of depression index, current registration date or 01 January 2023). Analyses were stratified by life decade and restricted to individuals with two or more MLTCs within each decade among both patients with depression (‘cases’) and their matched comparators. Patients contributed to all life-decades in which they had follow-up after index, restricted to periods of active CPRD registration. Burden indicators were defined within decade-specific windows rather than accumulated across follow-up, thereby reducing sensitivity to variation in prior observation time. We estimated decade-specific differences in MLTC prevalence and burden indicators by depression status. These analyses were cross-sectional and did not model incident MLTC accrual, but instead characterised the distribution of MLTC burden at each life stage.

A two-step clustering procedure was used separately within each life decade. First, Multiple Correspondence Analysis (MCA) was used to reduce the dimensionality of binary LTC indicators and capture latent patterns of disease co-occurrence. The number of dimensions retained was determined based on the point of variance stabilisation (elbow criterion from the scree plot). Second, k-means clustering was performed using the retained MCA coordinates. The optimal number of clusters was selected using the Calinski-Harabasz index, with the final solution additionally informed by the clinical interpretability of the resulting cluster profiles (eg, ‘Metabolic’ cluster characterised by high frequencies of type 2 diabetes and related complications). Further details about the metrics that informed the final cluster choice are available from the authors on request.

For each MLTC cluster, we estimated decade-specific mean overall burden and the prevalence of individual burden indicators. Associations between MLTC cluster membership and burden indicators were estimated using Poisson regression models with robust SEs, yielding incidence rate ratios (IRRs) representing proportional differences in mean burden indicator values. Analyses were stratified by depression status to assess differences between patients with and without depression. Overdispersion was assessed using dispersion diagnostics, and where substantial overdispersion was detected, negative binomial models were fitted. Logistic regression models were used for binary burden indicators. Both unadjusted and adjusted models were estimated, adjusting for age, sex, ethnicity and practice region. Since burden indicators were defined within fixed decade-specific windows rather than accumulated over follow-up, the analyses are less sensitive to variation in consultation frequency or prior observation time. Robust SEs accounted for clustering at the practice level. All analyses were conducted using R and Stata (V.18).

## Findings

Out of a total of 1 962 757 people with incident depression between 01 January 2003 and 31 December 2023, 1 563 065 (78%) had two or more LTCs recorded in their eletronic health record (EHR) during the follow-up period and were included in the analysis. In the comparator group for the same number of matched controls, 987 138 (or 50%) had two or more LTCs (table 1). The difference in MLTC between the two groups was larger in the early decades (decade 20: 69% vs 40%) and declined to late decades (decade 80: 96% vs 91%). The mean follow-up years after depression index was greater in patients with depression relative to their comparator group (14 years vs 10 years), that may partly reflect their younger mean age at study start compared with controls (37 years vs 42 years). The two groups were similar with respect to sex (61% women) and ethnicity (86% vs 85% of white ethnicity). Among both groups, the largest proportion of patients were from the North-West (23% vs 25%) and the South-East (22%) of England.

**Table 1:** Characteristics of people with and without depression and multiple long-term condition

	Depression n=1 563 065	No depression n=9 87 138
Follow-up (years, SD)	14 (6)	10 (7)
Age at cohort entry (years, SD)*	37 (20)	42 (24)
Gender		
Female	929 435 (61%)	602 367 (61%)
Ethnicity		
White	1 139 926 (86%)	676 348 (85%)
Black	36 114 (3%)	25 205 (3%)
Asian	72 601 (6%)	53 921 (7%)
Mixed	11 772 (1%)	7851 (1%)
Other	54 205 (4%)	30 735 (4%)
Region		
North East	58 308 (4%)	42 677 (4%)
North West	348 181 (23%)	244 584 (25%)
Yorkshire and Humber	48,232 (3%)	32 500 (3%)
East Midlands	36,859 (2%)	23 990 (2%)
West Midlands	264 859 (17%)	173 716 (18%)
East Anglia	55 505 (4%)	35 199 (4%)
London	220 143 (14%)	113 810 (12%)
South East	338 013 (22%)	216 139 (22%)
South West	159 037 (10%)	100 755 (10%)
Northern Ireland	6092 (1%)	3768 (0%)

All reported as N (%), aside from follow-up which are mean (SD).

* Patients with depression and comparators were individually matched on age within a 2-year window.

**Figure 1:**
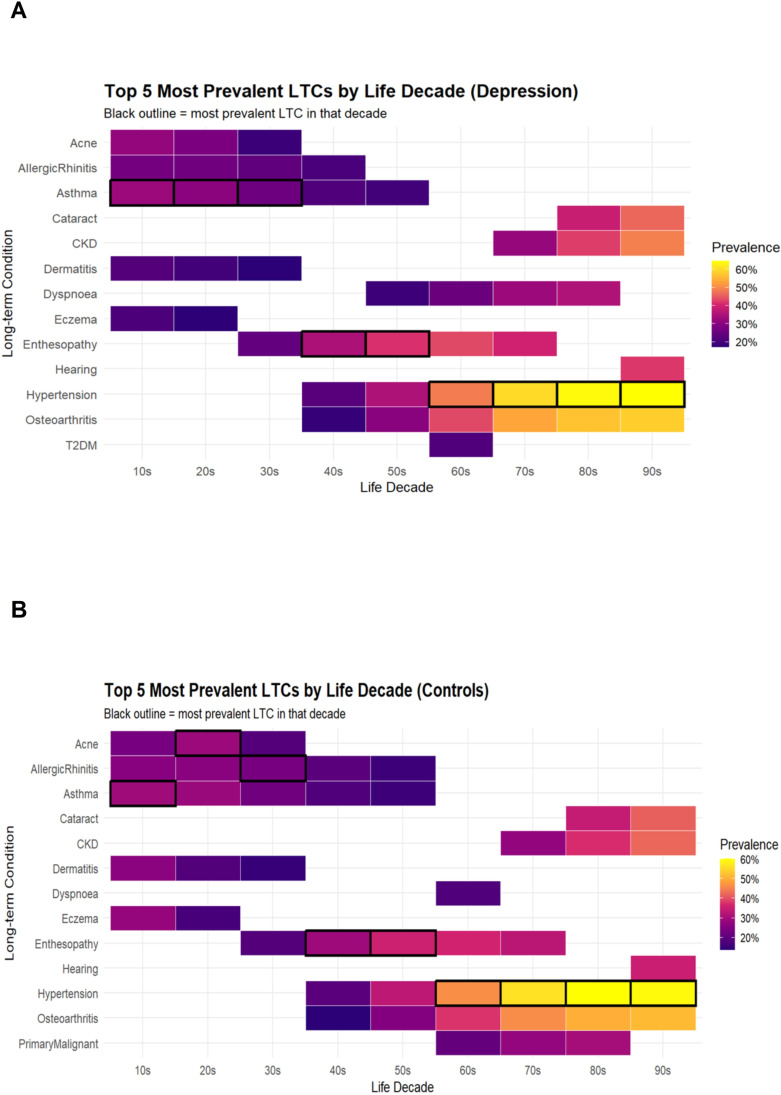
Most prevalent LTCs across the life decades for people with depression (A) and matched comparators (B). The intensity of the box indicates the decade-specific period prevalence of a specific LTC at each life decade. CKD, chronic kidney disease; LTC, long-term condition; T2DM, type 2 diabetes mellitus.

Figure 1 and online supplemental figure S2 illustrate distinct patterns in the top five and top 10 most prevalent LTCs across life decades between patients with depression and their matched comparators. Asthma emerged as the dominant LTC during the first three decades for patients with depression, whereas acne and allergic rhinitis were most common LTCs for the comparators. Patients with depression also experienced earlier and higher rates of type 2 diabetes within their top LTCs in mid-life. In contrast, sensory and hearing deficits emerged earlier within the comparators. Further, while both groups exhibited increasing prevalence of hypertension and osteoarthritis in later decades, these LTCs were more prevalent in depression. Such findings highlight accelerated and elevated MLTC accumulation in the depression cohort compared with comparators.

The number of MLTC clusters identified varied by life decade, ranging from four clusters in their 30s to nine clusters in the 50s, 60s and 90s among controls, with a similar range observed among cases. Exemplary cluster compositions are detailed in online supplemental figure S7, and cluster distributions by life decade are shown in online supplemental figure S3.

There were notable differences between patients with a depression history and their comparators in how MLTC patterns evolved across the life course. In the depression group, the atopic triad cluster dominated the early decades (10s–30s). Around mid-decades (40s–60s), however, patients with depression showed a more heterogeneous mix of multisystem, musculoskeletal (MSK), cardiovascular (CVD) and metabolic clusters, whereas the comparator group remained more heavily weighted towards metabolic low MLTC load clusters. In older decades (70s–90s), while both groups showed increasing cardiometabolic and MSK clusters, patients with depression experienced increased rates of multisystem and MSK clusters, implying a more complex and diffuse MLTC profile. Overall, patients with depression appeared to have patterns of high-burden MLTC clusters earlier relative to their counterparts.

### Patterning of burden indicators

**Figure 2:**
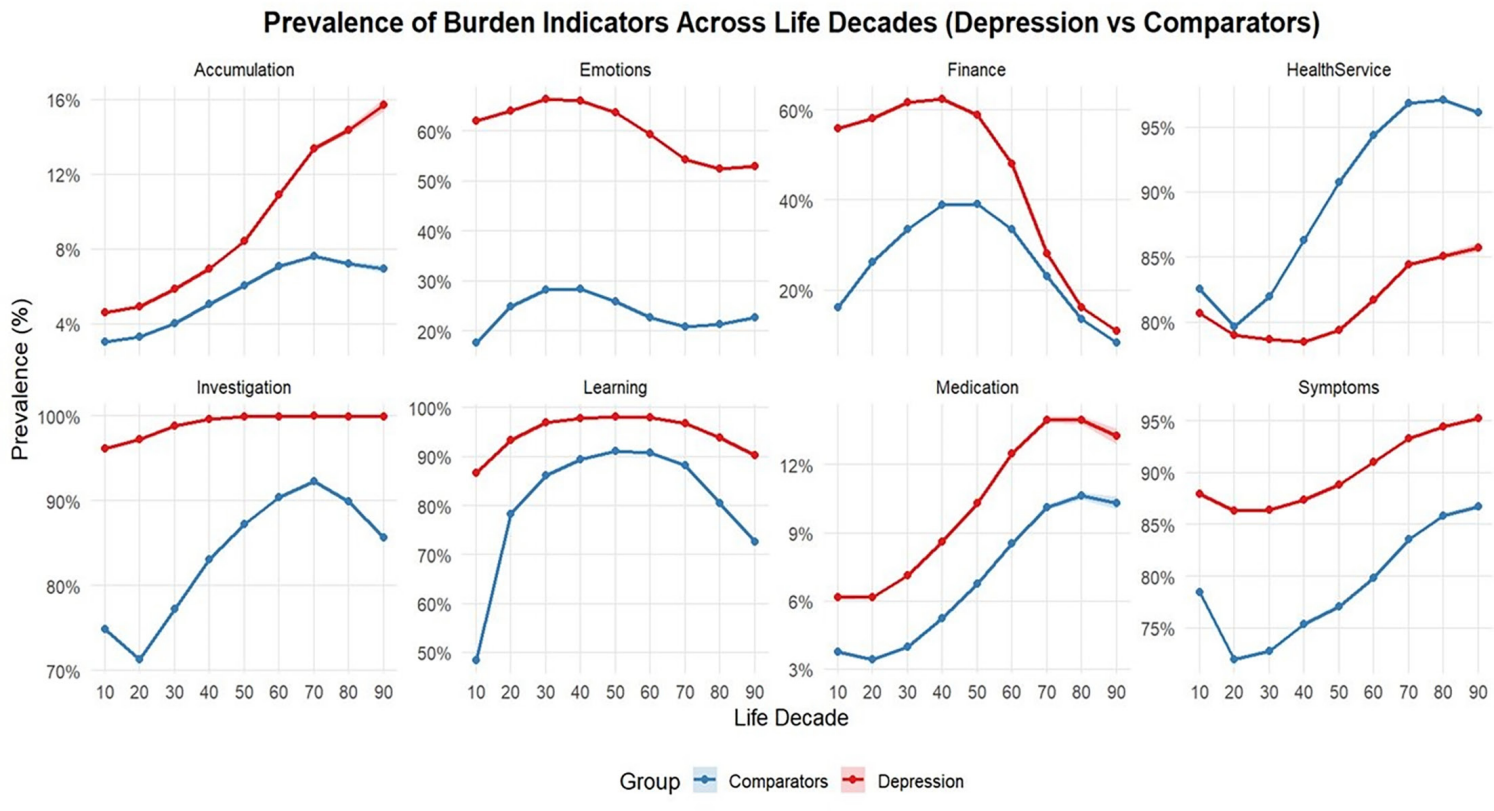
Patterning of burden indicators across the life decades for people with depression and matched comparators. The lines indicate the % of patients at each life decade that had a recording of a specific burden indicator.

Figure 2 illustrates the trends of the eight burden indicators over time at each life decade. As anticipated, patients with depression consistently showed higher burden relative to their comparators. Specifically, investigation and monitoring, learning and adapting indicators remained high (80–90%) across most decades for both groups. Symptom burden, however, revealed a steeper increment with age in patients with depression. Financial burden was most pronounced in early decades and declined after midlife, with patients with depression having higher levels throughout. For comparators, health service and administration burden dominated in late life decades (50s–90s), while learning and adapting were highest in early decades.

### Dominant clusters by life decade-specific overall and specific burden

**Figure 3:**
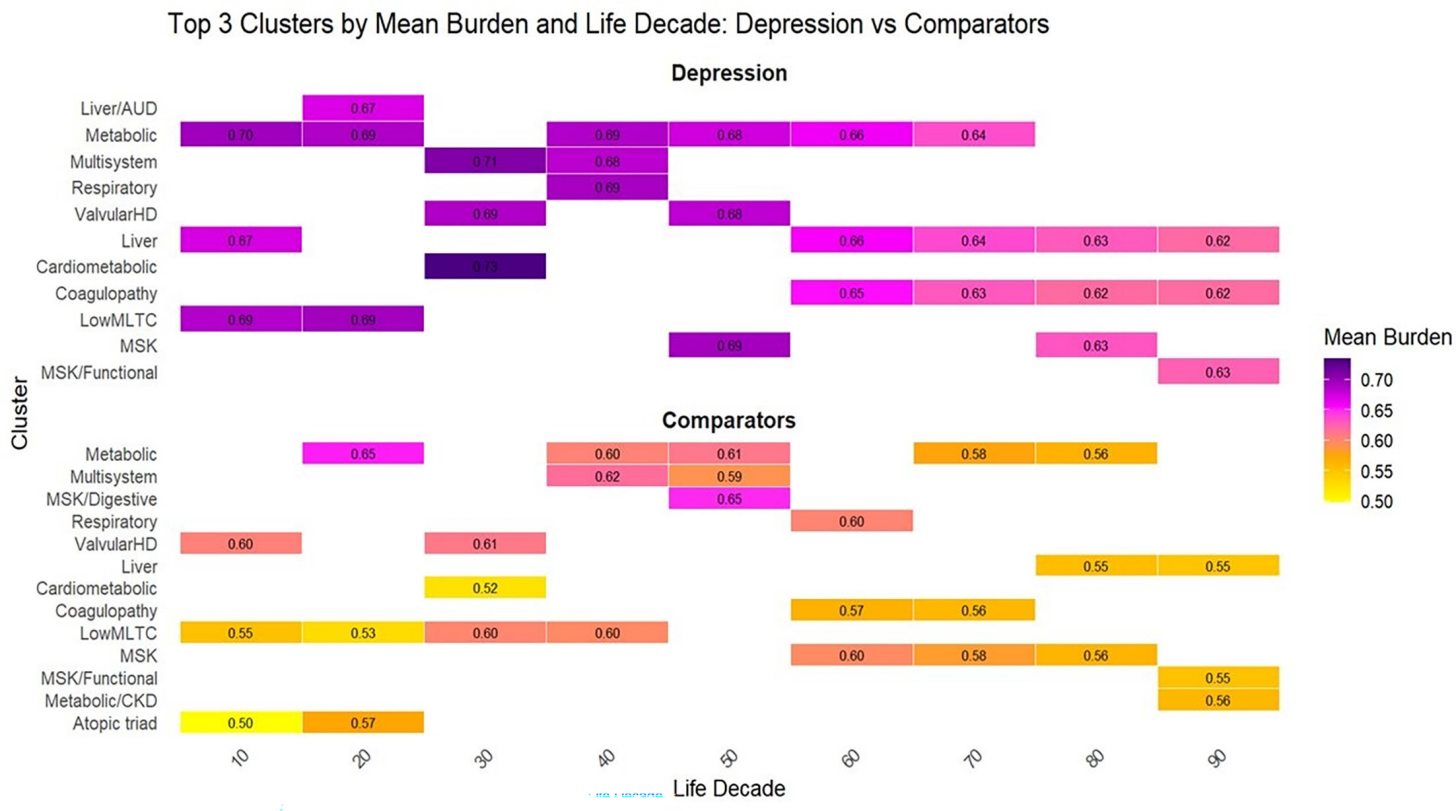
Top 3 clusters in terms of number of burden indicators across the life decades for people with depression (top graph) and matched comparators (bottom graph). Boxes indicate the overall (mean) number of burden indicators that patients in each cluster had at each life decade. CKD, chronic kidney disease; MLTC, multiple long-term conditions; MSK, musculoskeletal; AUD, alcohol use disorder; HD, heart disease.

Regarding the overall burden across life decades (figure 3), the findings exposed higher multidimensional burden patterns in depression, with disparities emerging by the third decade and widening thereafter. The higher mean burden value (a higher average count per patient) indicated more pervasive impact on both patient-centred and system-centred burden. Several clusters (liver/alcohol use disoder (AUD), metabolic, multisystem) reached mean burden values of 0.60–0.70 in early decades 20s–40s, indicating that patients experienced multi-domain burden simultaneously. The comparator group, in contrast, had lower average burden (0.50–0.55).

Similar complex patterns were observed with respect to dominant clusters of specific burden indicators (online supplemental figure S4). Investigation and monitoring and medication-related burdens were substantially higher among patients with depression across most life decades. For example, medication burden affected 15% of patients with depression versus 2% of comparators, while 40% of patients with depression had a recorded investigation and monitoring indicator in their 10s compared with only around 10% of those without depression.

### Impact of clusters of MLTCs on overall and specific burden indicators

Next, we examined decade-specific associations between MLTC clusters and overall burden (online supplemental figure S5). Across most decades, patients with depression experienced higher mean counts of MLTC-related burden indicators than their matched controls, whereas comparators showed a sharper increase in early decades followed by a more stable pattern later in life. In this cross-sectional context, the adjusted IRRs (aIRRs) represent proportional differences in mean burden counts. For example, an aIRR of 1.02 (95% CI 1.01 to 1.03) for the metabolic cluster (eg, type 1 diabetes, type 2 diabetes, diabetic retinopathy) in their 10s indicates that patients with depression had approximately 2% higher mean overall burden than controls. This difference increased with age, rising to 1.14 (95% CI 1.12 to 1.15) for the respiratory cluster (eg, interstitial lung disease, pulmonary fibrosis) in the 40s, 1.16 (95% CI 1.16 to 1.19) for the CVD cluster (eg, ischaemic heart disease, hypertension, angina) in the 60s, and 1.17 (95% CI 1.16 to 1.18) for the MSK/functional cluster (eg, osteoarthritis, enthesopathy, hearing loss) in the 90s. Logistic regression analyses for individual indicators showed patterns consistent with the Poisson/negative binomial results (online supplemental figure S6), with patients with depression generally showing a higher likelihood of specific MLTC-related burdens than their matched controls. Overall, less prevalent clusters such as metabolic, respiratory and liver/AUD showed comparatively high levels of patient-centred burden and healthcare utilisation, while investigation and learning burdens remained high across life stages and financial strain was most marked in early decades.

## Discussion

Leveraging prospective data from one of the world’s largest primary care EHR datasets, the study documented substantially higher prevalence of MLTC in patients with a history of depression relative to those without any depression history. Distinct clusters of MLTC emerged, reflective of empirical patterns of disease accumulation in the real world, indicating that LTCs accumulate and cluster in non-random ways across life decades. Using novel patient-centred burden indicators, the study mapped how these clusters translated into overall and domain-specific burden, revealing systematic differences between and within groups with and without a history of depression across life decades. The consistently higher, earlier, and broader burden in patients with depression imply an accelerated accumulation of multidomain burden. The findings help substantiate depression as a key factor for patient-centred and system-centred burden, reinforcing the need for tailored and psychosocially informed life-stage adapted MLTC care. Early-life interventions targeting specific conditions combinations (eg, atopic triad, metabolic disorders) may be valid targets to help prevent the emergence of the burdensome MLTC patterns seen in the older age adults in this study.

Novel findings emerged with regards to the accumulation of burden indicators within specific life decades and clusters. Atopic conditions, though common, were not the primary drivers of overall burden. In contrast, less prevalent clusters (eg, metabolic, respiratory, liver/AUD) were associated with high rates of patient-centred burden and healthcare utilisation. Such distinctions are of practical value for wider priorities in healthcare systems where shared service improvements may be applicable across clusters of conditions.^18^ They also align with the evidence that fragmented care pathways and deficient care coordination may exacerbate the burden of MLTC.^19 20^ Embedding system-wide reforms alongside targeted interventions is critical to improving outcomes and reducing inequalities for patients living with depression and co-occurring LTCs. This suggestion aligns well with current care practices that often provide CVD or metabolic screening for patients on treatment but may miss those with milder symptoms.^21^ Moreover, the burden of underlying conditions is rarely systematically addressed in routine consultations, such as in the quality and outcomes framework indicators for mental health.

Burden-specific patterns are also notable. While investigation and learning burdens remained high across most life stages, financial strain was most pronounced in early decades. The latter aligns well with the large economic burden of, for instance, diabetes and its complications in the UK (≈£10.6 billion direct cost plus £3.3 billion indirect).^22^ Emotional burden was also consistently increased in patients with depression, underlining the ongoing psychosocial stress of mental and physical MLTC. This finding substantiates the need to integrate evidence-based psychosocial interventions, such as National Health Service (NHS) Talking Therapies (NHS TT), within the management of physical LTCs to address their cumulative and mutually reinforcing burden. Current evidence in this area remains, however, limited and shows only modest benefits.^23 24^

Our findings add to a growing body of evidence on the intersection of depression and MLTC.^2^ Previous work has shown that MLTC was associated with increased healthcare utilisation and costs in the UK context.^25^ Systematic reviews of existing interventions for patients with MLTC suggested that the evidence remains of low certainty and effects on mental health outcomes are modest at best.^26^ Besides, evaluations of NHS TT effectiveness indicated that the nature and number of physical LTCs reduce the likelihood of improvement in therapy outcomes.^24^ The present study corroborates this evidence by demonstrating that not only is MLTC more common in depression, but that clusters of conditions differ by life-stage and carry differential burden in terms of symptoms, service‐use and functional abilities. It underlines, therefore, how patient-centred burden indicators (though only partially captured in EHR) allow novel insight into the lived experience of MLTC and reinforce that interventions cannot assume all patients with MLTC share the same needs.^3^

### Strengths and limitations

Key strengths of this study include the scale and representativeness of CPRD, the inclusion of over 250 validated LTCs, and the use of patient-informed burden metric reflecting a nuanced, person-centred perspective on MLTC.^27^ The life-course framework enabled examination of MLTC patterns across decades, capturing emerging life-course patterns rarely evidenced in previous studies. Several limitations should also be noted. First, the study has not aimed to demonstrate causal associations between MLTC clusters and person-centred burden. As such, the findings preclude any inferences regarding the temporality of associations between MLTC clusters and burden indicators (eg, some burden may precede or follow the onset of specific conditions). Similarly, as follow-up is limited by the extent of EHR data available, we can only capture a subset of each person’s life course (eg, a maximum of ~20 years). This work may therefore reflect different pathways and patterns across the life course (eg, burden and MLTC for older adults with first depression code at age 60 years) and differential coding practices by primary care practitioners across time. Nonetheless, many burden indicators are inherently related to underlying disease or treatment corollaries, such as breathlessness in respiratory disease or falls from antihypertensives. Second, patients with depression had a modestly longer follow-up period relative to their comparators, consistent with CPRD evidence for people living with other chronic conditions.^28 29^ These mechanisms may partly contribute to the observed difference in MLTC prevalence, but they are unlikely to account for the large gap observed (78% vs 50%). Third, CPRD provides rich clinical information, yet it captures only a subset of patient-centred burden indicators, omitting important aspects of lived experience (eg, housing, quality of life, work). These are either incompletely and inconsistently recorded in EHRs or embedded in free text that is unavailable for research. The findings of the present study would benefit from confirmation with self-reported data from cohort studies with rich repeated psychosocial and environmental variables. Fourth, differential engagement with the care system (eg, screening programmes, annual reviews) may further exaggerate burden differences if patients with depression have more recorded events through more frequent contact. Fifth, we have not fully explored drivers and variance of these associations here. Future exploration of patterns by gender, ethnicity (both for broad ethnicity categories and for more specific coding), deprivation and other confounders may highlight specific patterns within groups and help direct targeted intervention. Lastly, while CPRD is broadly representative of the UK primary care population, it may be limited in its generalisability to populations outside the UK and also of populations that are less likely to access primary care services, such as healthy young adults.

## Clinical implications

MLTC is highly prevalent among patients living with a history of depression and unfolds along distinct, increasingly burdensome condition profiles across the life course. Different clusters tend to dominate at different ages, shaping lived experience and placing substantial pressure on individuals and the healthcare systems. We have identified, for instance, early-life clusters (eg, atopic triad, metabolic disorders), marked by inflammatory mechanisms that may represent promising modifiable pathways for prevention or early intervention of burdensome MLTC, such as enhanced care pathways for young people presenting with these conditions, particularly for young people with depression. Digital health and integrated care models offer scalable opportunities to help support patients, but future research is needed to develop, evaluate and implement co-designed, multicomponent interventions that address depression and co-occurring LTCs identified here. Advancing this agenda is critical to reduce cumulative lifetime burden and improve patient-centred outcomes among the growing number of patients living with common mental health disorders.

## Supplementary material

10.1136/bmjment-2026-302705 online supplemental file 1

## Data Availability

Data may be obtained from a third party and are not publicly available.
