# Supplementary material for: Life-course patterning of MLTC clusters and their patient-centred burden in depression: a population-based study using real-world data
Source: BMJ Ment Health. 2026 Jul 21;29(1):e302705. doi: 10.1136/bmjment-2026-302705 (PMC13404816; doi:10.1136/bmjment-2026-302705)
Supplement: online supplemental file 1 [file bmjment-29-1-s001.docx]

**Supplementary material**

**Supplementary methods**

**Definition of burden indicators**

The burden indicators were initially described in an extensive qualitative assessment with people living with multiple long-term conditions.^1^ These concepts were then built on through a multi-step process to identify EHR based codes for each one, which is detailed elsewhere.^2^ Briefly, one member of the team with extensive experience of clinical coding in primary care as a General Practitioner (GP) searched SNOMED CT, a structured clinical vocabulary for use in EHRs as a first step to identify codes for each of the outlined burden areas.

Emerging codelists were then reviewed with a second member of the team before being sent to a second clinical reviewer within the team who undertook a verification process of each code, considering whether the code list correctly reflected the concept being captured and whether other potential codes were known that should be added.

For concepts not represented by clinical code list, there was some engineering required. For example, ‘numbers of GP appointments’ within the ‘health service and administration’ theme of work, would not be reflected by a clinical code list but can be calculated by counts within the relevant field within EHRs.

The 8 themes included ‘learning and adapting’ (learning about new and existing conditions and their management, including the physical and psychological adjustments required), ‘accumulation and complexity’ (the additional and cumulative burden of living with multiple, rather than just one, long-term condition), ‘investigation and monitoring’ (the work of tests related to MLTCs), ‘health service and administration’ (work related to navigating health services) ‘medication work’ (work associated with taking and managing medications), ‘financial work’ (the financial impact of living with MLTCs), ‘symptom work’ and ‘emotional work’.

For ‘Medications’, a single prescription was not considered sufficient for an individual to be included in the medication burden. To reflect chronicity of medication use, an individual needed to have at least one prescription for a specific medication in at least three out of four quarters of a year.

**References**

1. Holland E, Matthews K, Macdonald S, et al. The impact of living with multiple long-term conditions (multimorbidity) on everyday life - a qualitative evidence synthesis. *BMC Public Health* 2024;24(1):3446. doi: 10.1186/s12889-024-20763-8
2. Fraser SDS, Holland E, Laidlaw L, Francis NA, Macdonald S, Mair FS, Alwan NA, Boniface M, Hoyle RB, Fair N, Dylag JJ, Shiranirad M, Chiovoloni R, Stannard S, Poole R, Akbari A, Ashworth M, Dregan A. Capturing the human impact of living with multiple long-term conditions in routine electronic health records - lost in translation? J Multimorb Comorb. 2025; 1;15:26335565251329869.


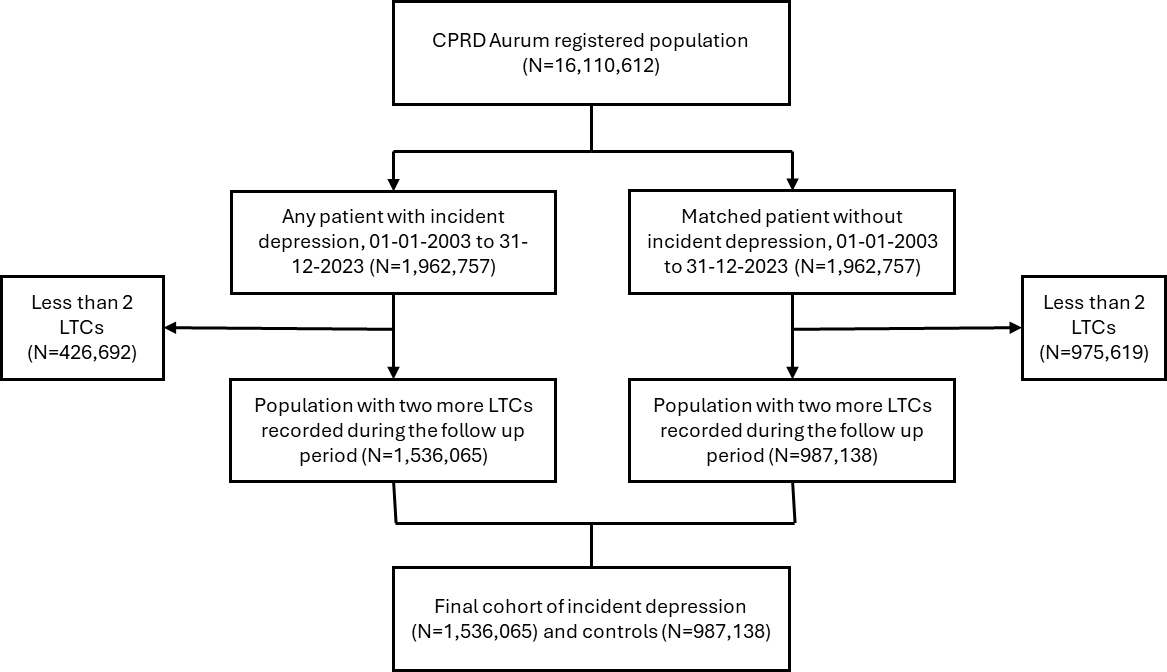
**Figure S1. Patient and control inclusion flowchart**

Table S1. List of Long-Term Conditions in the Dataset (N=263)

| Abdominal hernia | Acne |
| --- | --- |
| Addison disease | ADHD |
| Alcohol liver disease | Agranulocytosis |
| Aldosteronism | Alcohol use disorder |
| Alopecia areata | Allergic rhinitis |
| Alzheimer disease | Amyotrophic lateral sclerosis |
| Amnesia | Age-related macular degeneration |
| Angina | Anaphylaxis |
| Antiphospholipid | Ankylosing spondylitis |
| Aortic aneurysm | Aphasia |
| Aplastic anemia | Appendicitis |
| Arrhythmia | Arterial disease |
| Asbestosis | Asplenia |
| Asthma | Ataxia |
| Atrial fibrillation | Autism |
| Autoimmune liver | Autonomic neuropathy |
| Barrett | Bells Palsy |
| Bipolar disorders | Bronchiectasis |
| Cachexia | CAD |
| Cardiomyopathy | Carpal Tunnel |
| Cataract | Cerebral palsy |
| Chronic fatigue syndrome | Cholelithiasis |
| Cholangitis | Chronic liver |
| Chronic cystitis | Chronic pancreatitis |
| Chronic pain | Cirrhosis |
| Chronic sinusitis | Coagulopathy |
| Chronic kidney disease | Collapsed vertebra |
| Coeliac disease | Congenital septal defect |
| Congenital heart disease | Cranial palsy |
| COPD | Cushing syndrome |
| Crohn disease | Delusional disorders |
| Cystic fibrosis | Dermatitis |
| Depersonalisation | Dissociative disorder |
| Diabetic nephropathy | Downs syndrome |
| Diverticular disease | Deep venous thrombosis |
| Diabetic retinopathy | Dysmenorrhea |
| Dyslipidemia | Dystonia |
| Dyspnea | Eczema |
| Eating disorder | Encephalitis |
| Ehlers Danos | Endometrial hyperplasia |
| Endocrine | End stage CKD |
| Endometriosis | Epilepsy |
| Enthesopathy | Fibromatosis |
| Erectile dysfunction | Fronto-temporal dementia |
| Fibromyalgia | Gastroesophageal reflux disease |
| Gastritis | Gingivitis |
| Giant Cell arteritis | Glaucoma |
| Glomerulonephritis | Gout |
| Hereditary angioedema | Haemochromatosis |
| Haemophilia | Haemorrhagic stroke |
| Hearing problems | Heart failure |
| Heart valve disorder | Hemiplegia |
| Hepatic failure | Hepatitis B |
| Hepatitis C | Hepatitis |
| Herpes Zoster | Hidradenitis |
| HIV | Henock Schonlein Purpura |
| Huntington’s disease | Hyperkalaemia |
| Hypertension | Hyperthyroidism |
| Hypoglycaemia | Hypogonadism |
| Hypo-Hypersplenism | Hypokalaemia |
| Hypotension | Hypothyroidism |
| Inflammatory bowel disorder | Irritable bowel syndrome |
| Iron deficiency anemia | Ischemic heart disease |
| Interstitial lung disease | Immunodeficiency diseases |
| Inflammatory eye disorder | Insomnia |
| Intellectual disability | Intervertebral disc disorder |
| Idiopathic pulmonary fibrosis | Ischemic stroke |
| Leiomyoma | Lewy body dementia |
| Lichen planus | Liver |
| Lymphoma | Lymphoedema |
| Malnutrition | Malaria |
| Meniere disease | Marfan syndrome |
| Mononucleosis | Myocardial infarction |
| Muscular dystrophy | Motor neuron disease |
| Myasthenia Gravis | Multiple sclerosis |
| Myelitis | Non-alcoholic fatty liver disease |
| Neuralgia | Nasal polyps |
| Neuropathy | Neuropathic bladder |
| Non-rheumatic valve disorder | Neutropenia |
| Obstructive uropathy | Obesity |
| Oedema | Obsessive compulsive disorder |
| Obstructive sleep apnea | Oesophageal varices |
| Osteoarthritis | Osteoporosis |
| Other Liver disorders | Other haemolytic anaemia |
| Paraplegia | Peripheral artery disease |
| Parkinson Disease | Pancreatitis |
| Pulmonary embolism | Parathyroidism |
| Peptic ulcer | Polycystic ovary syndrome |
| Periodontal | Pemphigus |
| Peritonitis | Pericarditis |
| Personality disorder | Peripheral neuropathy |
| Pleural effusion | Pernicious anemia |
| Pneumothorax | Pituitary Disease |
| Polymyalgia rheumatica | Pneumonitis |
| Posterior uveitis | Polycythemia vera |
| Primary Thrombocytopenia | Polyneuropathy |
| Psoriasis | Prostatism |
| Psychotic disorders | Psoriatic Arthritis |
| Post-traumatic stress disorder | Ptosis |
| Pulmonary fibrosis | Pulmonary other |
| Rheumatic arthritis | Pulmonary heart disease |
| Reactive arthropathy | Primary malignant disease |
| Retinal disorders | Raynaud syndrome |
| Rhabdomyolysis | Respiratory failure |
| Rheumatic valve disorder | Retinal vascular occlusion |
| Sarcoidosis | Rheumatic heart disease |
| Scleritis episcleritis | Rosacea |
| Scoliosis | Schizophrenia |
| Schizoaffective disorders | Scleroderma |
| Sensory disorders | Secondary Thrombocytopenia |
| Sjogren syndrome | Sickle Cell disease |
| Spina Bifida | Systemic lupus erythematosus |
| Spondylolisthesis | Spinal stenosis |
| Substance use disorder | Spondylosis |
| Type 1 Diabetes Mellitus | Supraventricular tachycardia |
| Tachycardia | Type 2 Diabetes Mellitus |
| Thrombocytopenia | Thalassemia |
| Thyroid disorder | Thrombophilia |
| Thyrotoxicosis | Thyroiditis |
| Tinnitus | Transient Ischemic Attack |
| Tuberculosis | Transversal myelitis |
| Turners | Tubulo-interstitial disease |
| Ulcers | Ulcerative Colitis |
| Urolithiasis | Urinary incontinence |
| Vascular dementia | Uveitis |
| Vasculitis | Valvular heart disease |
| Visual Impairment | Venous thromboembolism |
| Vitamin deficiency | Vitamin B12 deficiency |
| Viral hepatitis | Vitiligo |

*Note*: The definition of each long-term condition is exclusive (no overlapping medical codes). For instance, inflammatory bowel disorders includes those medical codes that could not be classified as Crohn or Ulcerative colitis disorders.


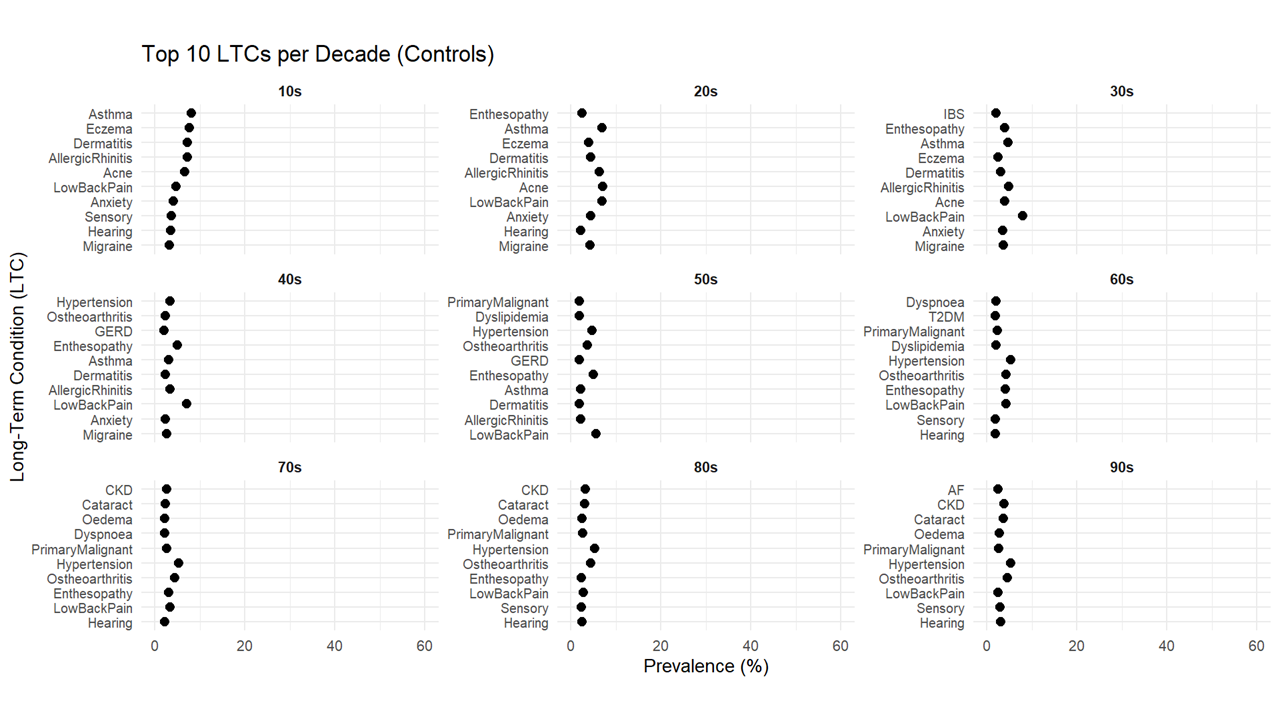

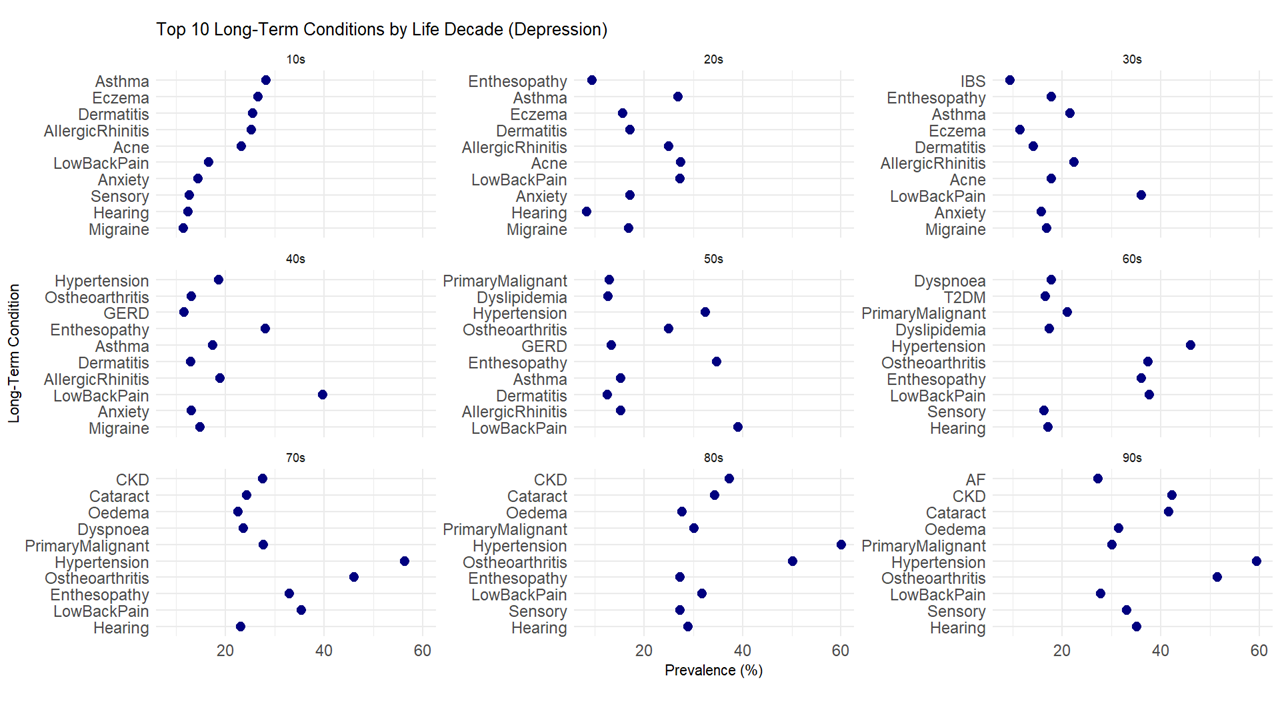


**Figure S2. Plots describing the distribution of the ten most common long-term conditions among cases and controls at each life decade.**


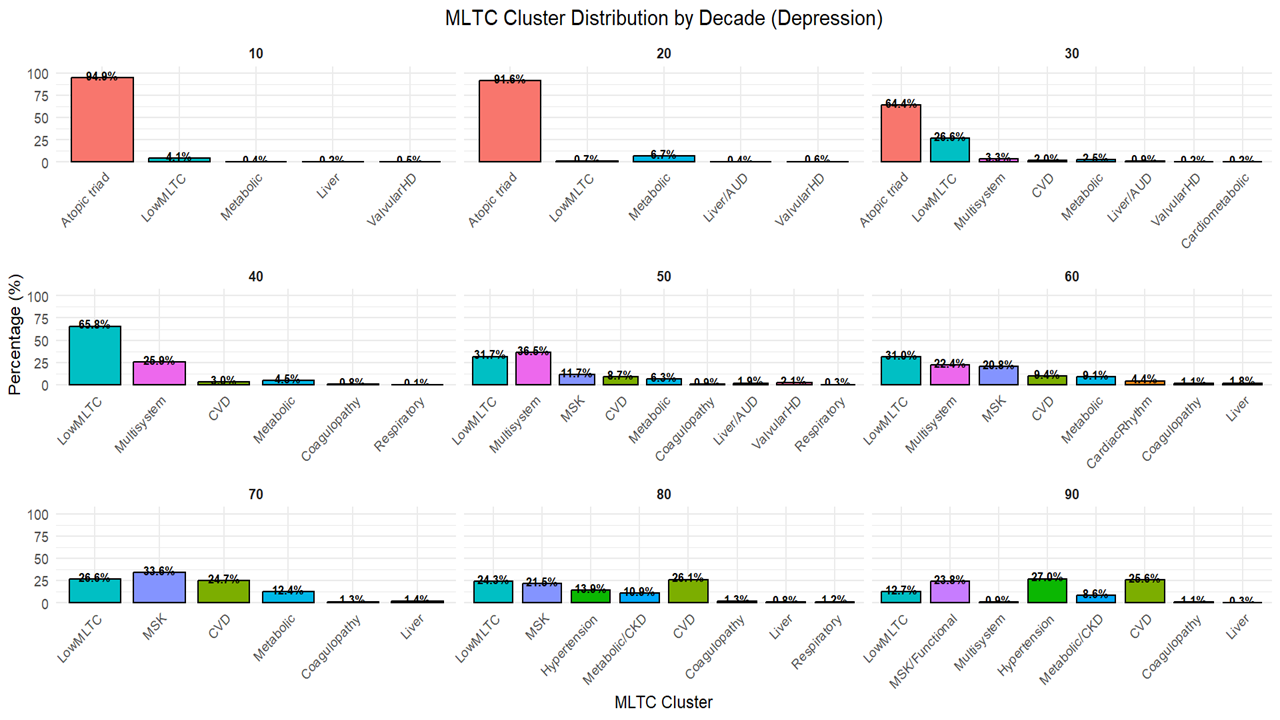


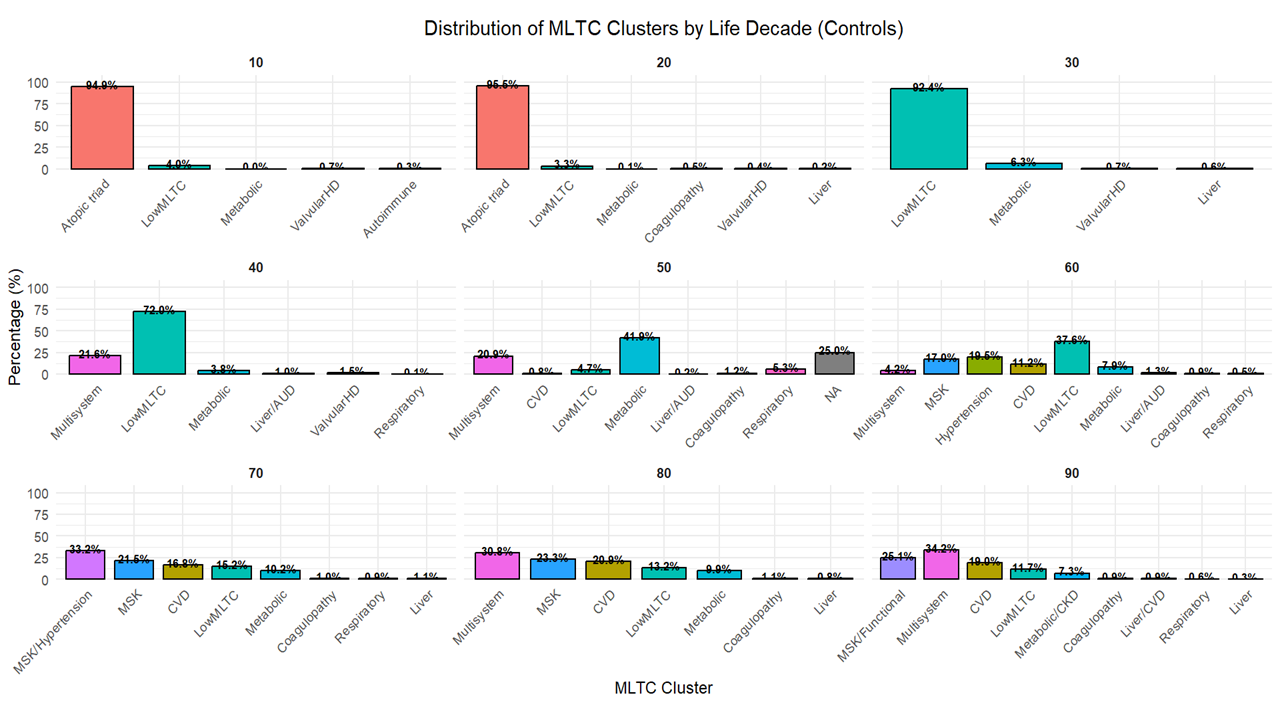


**Figure S3. Distribution of MLTCs clusters across the life decades for people with depression (top panel) and matched comparators (bottom panel).** Bar charts indicate the % of patients at each life decade that belonged to a specific cluster. AUD=Alcohol use disorder; Valvular HD- Valvular Heart Disease; MSK=Musculoskeletal; CVD=Cardiovascular disorders; LowMLTC=low multiple long term conditions; CKD=Chronic kidney disease


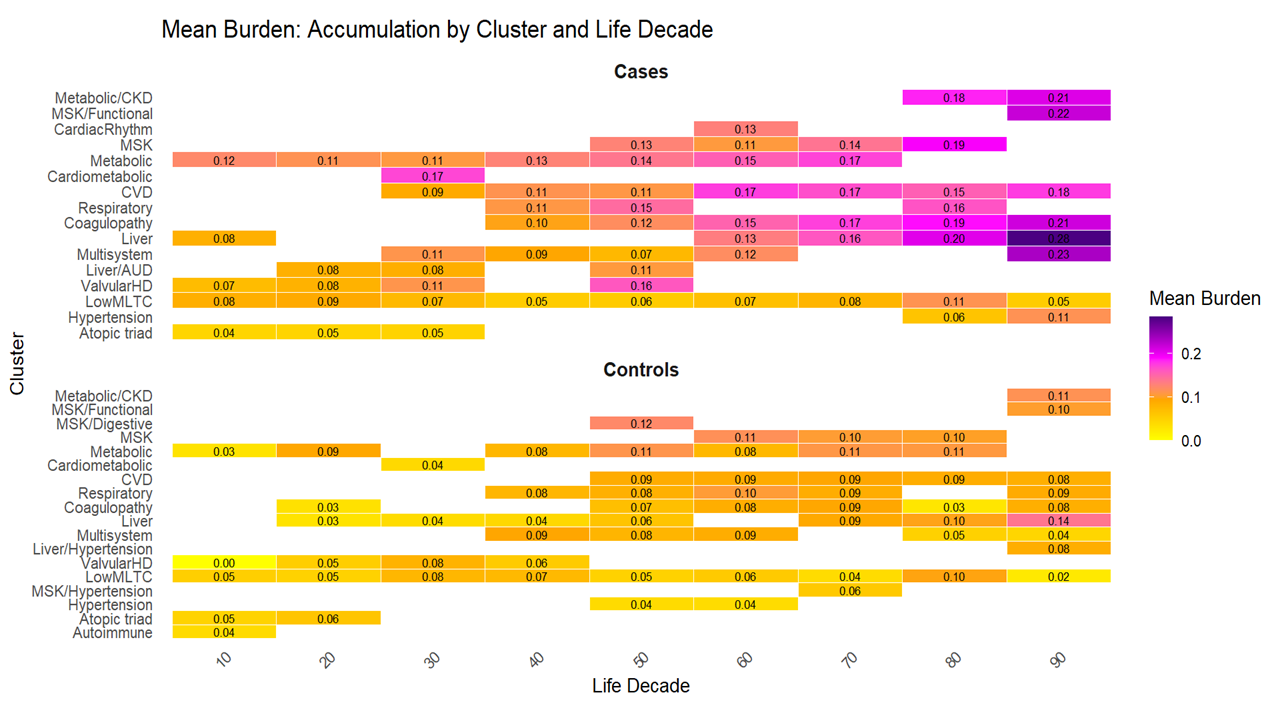


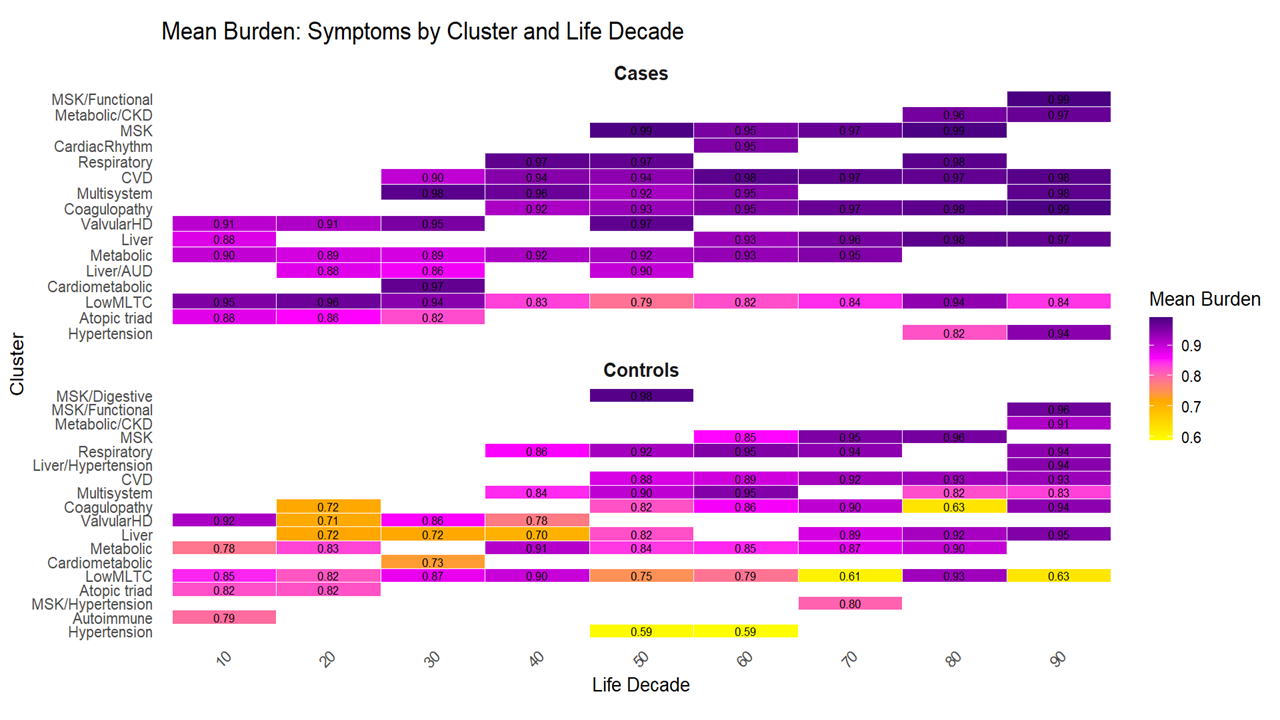


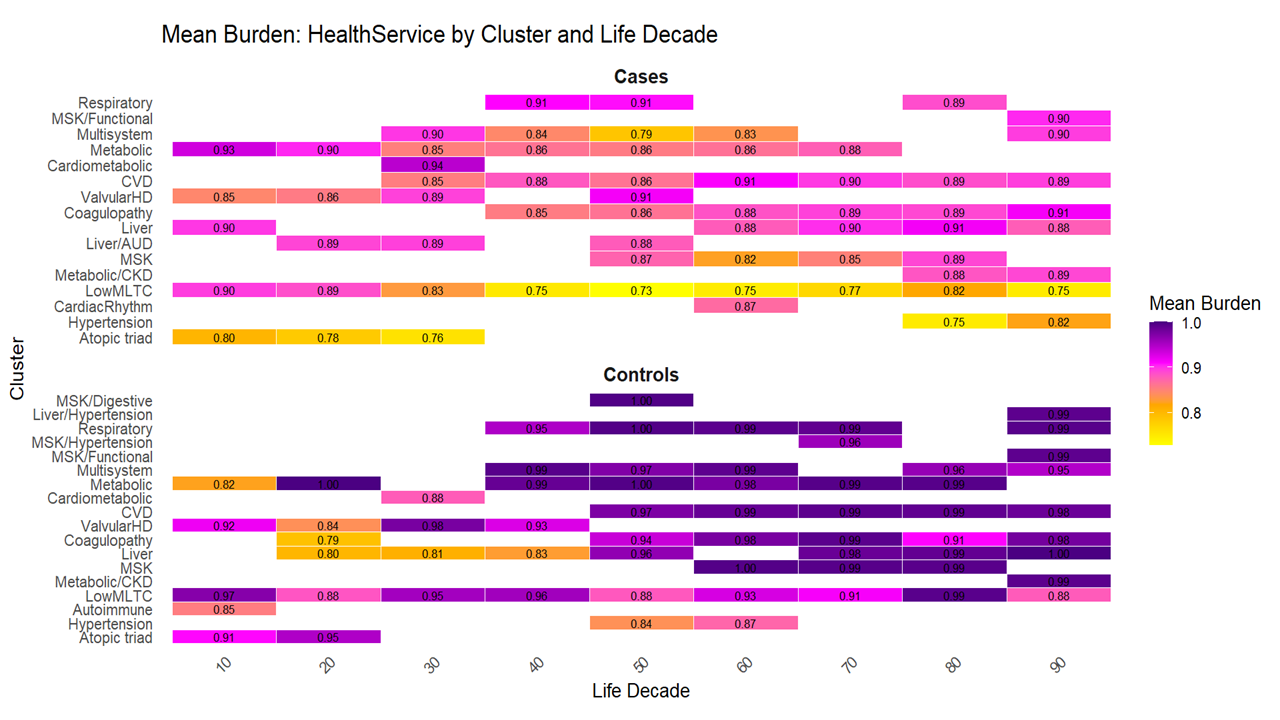


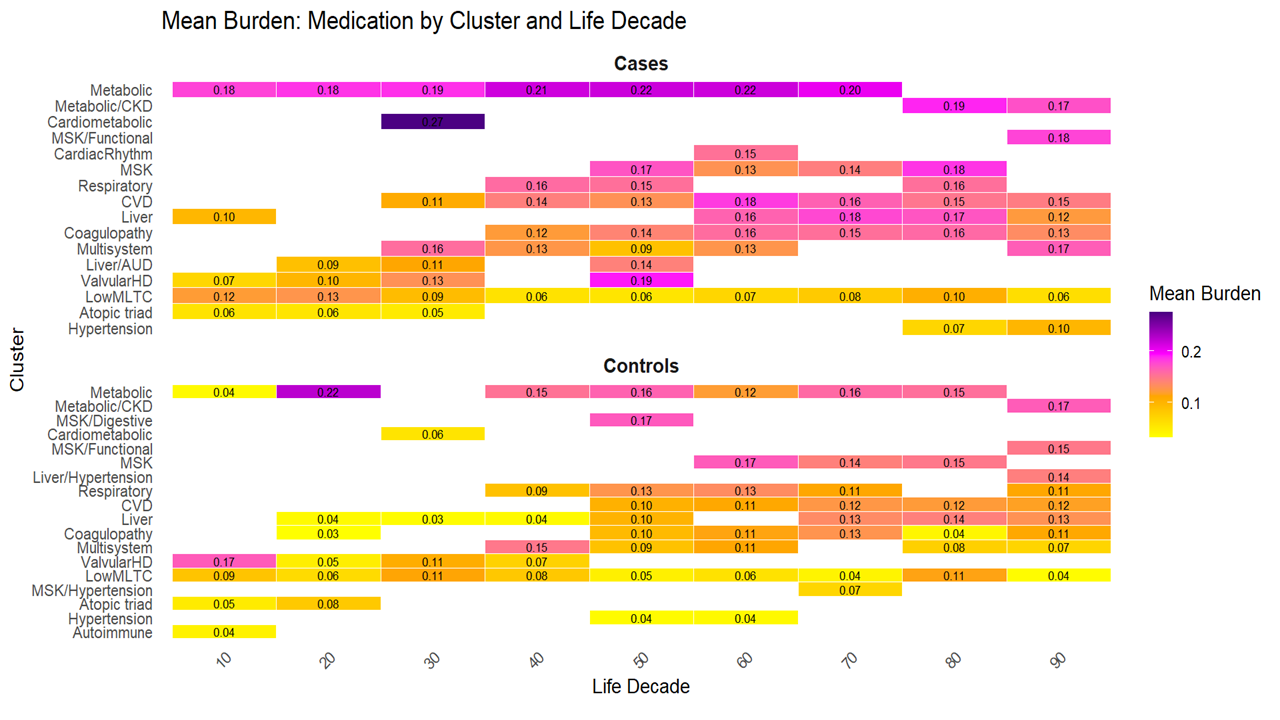


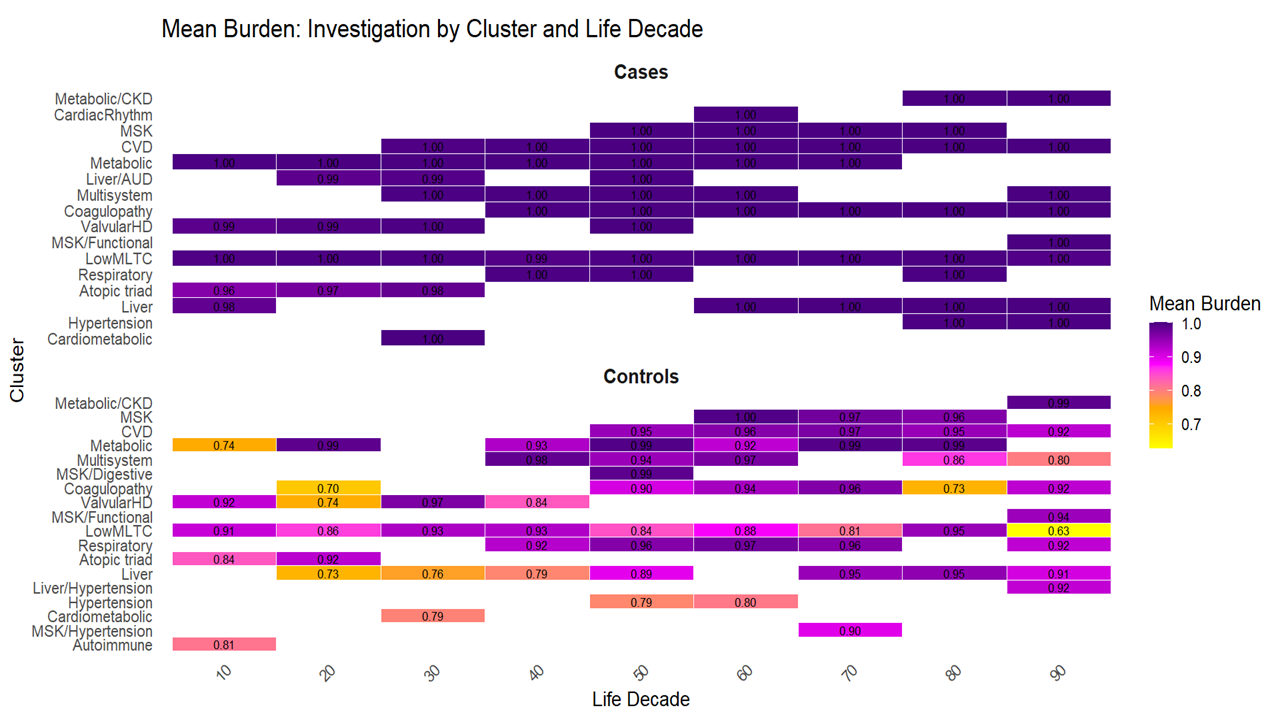


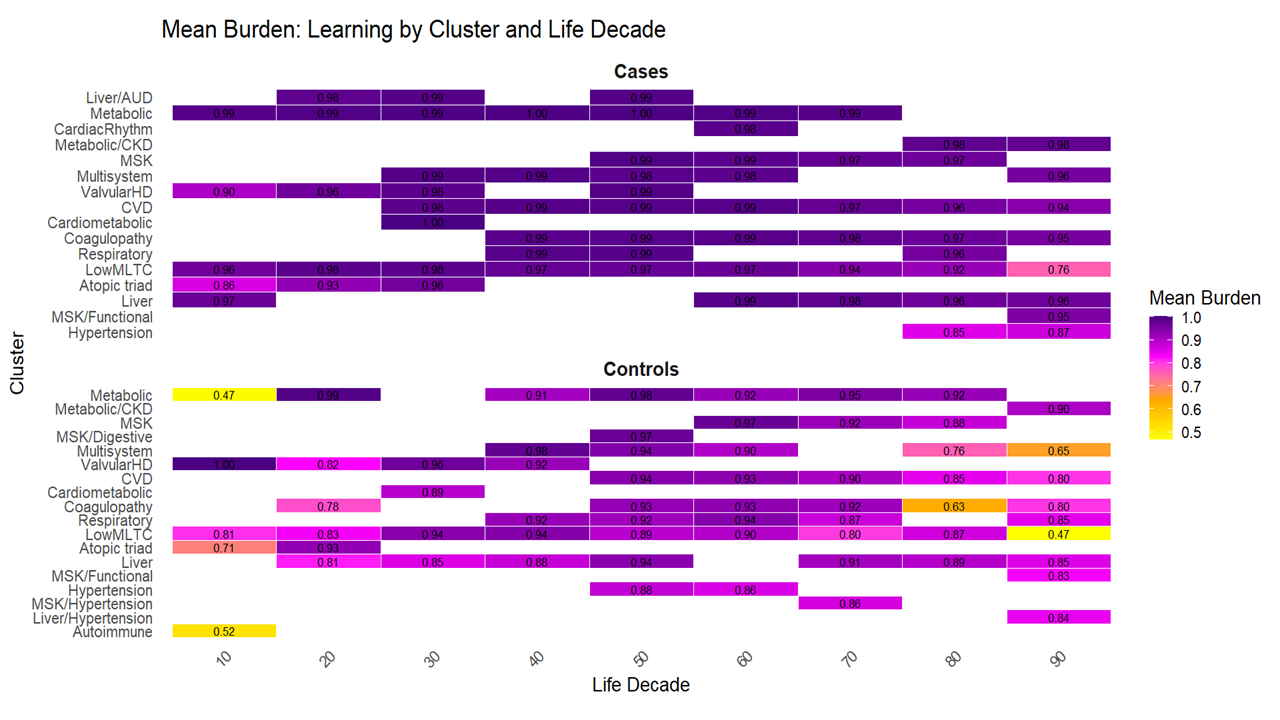


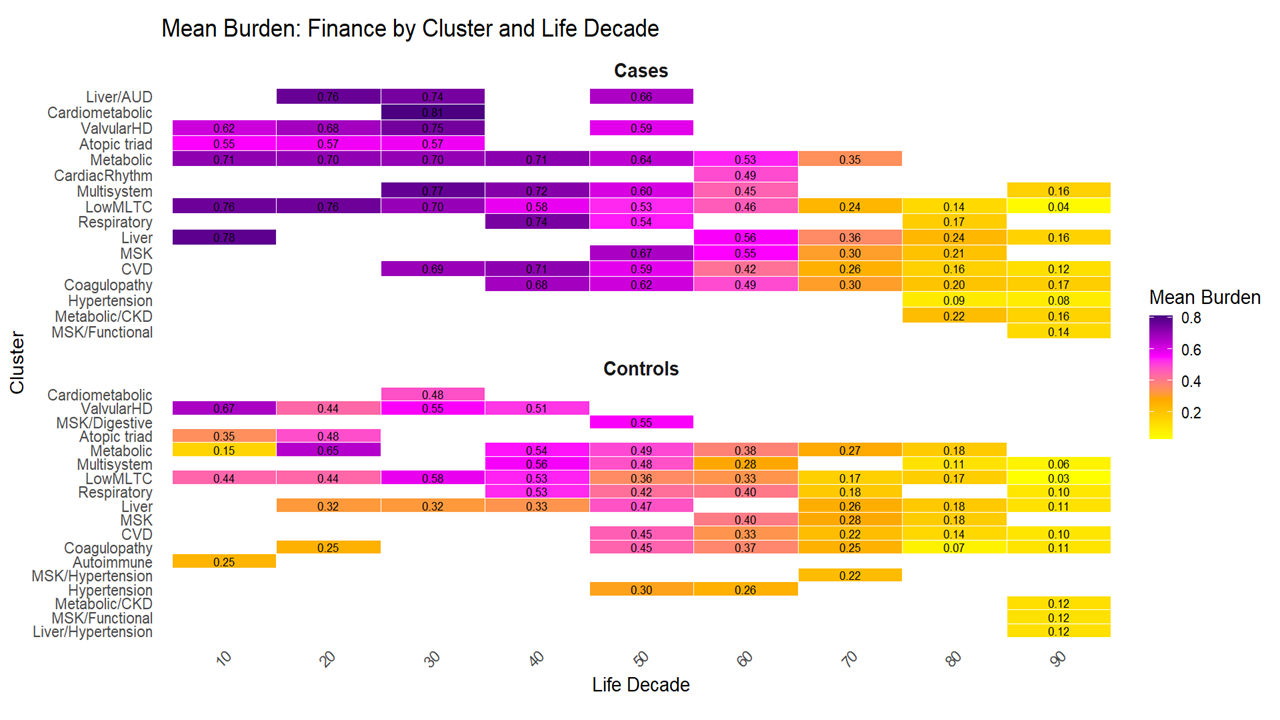


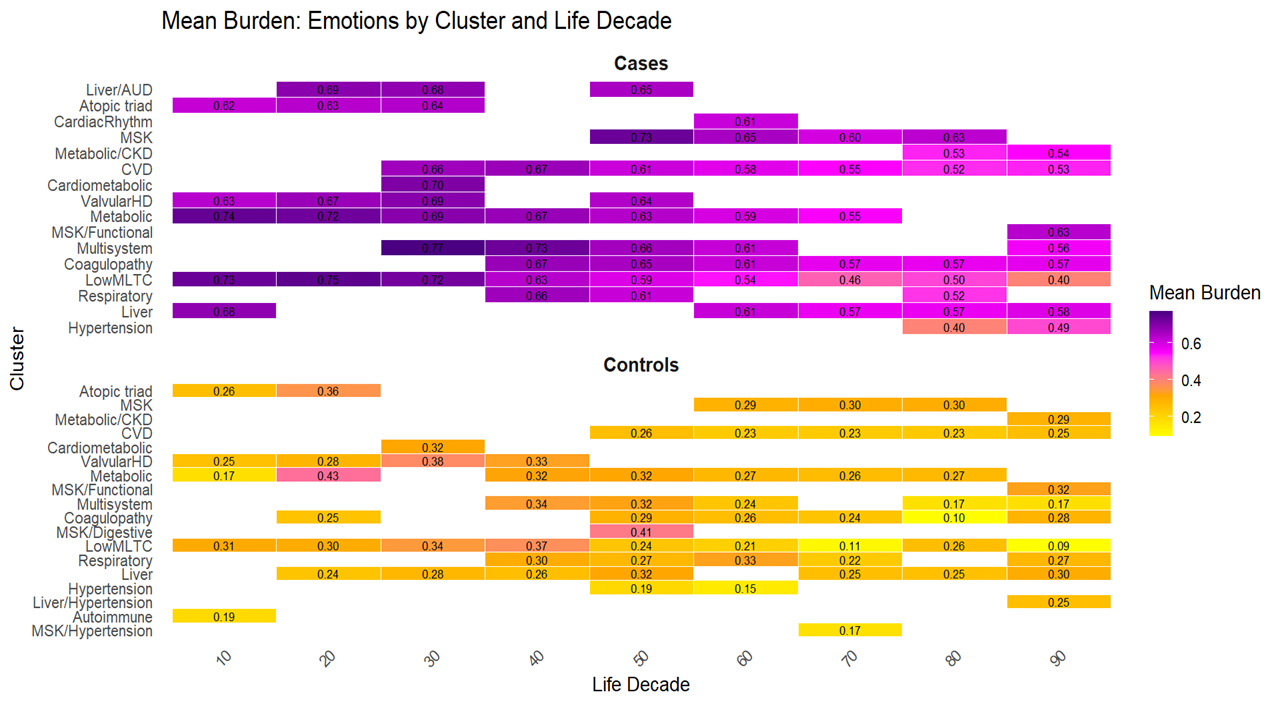


**Figure S4. Plots describing the distribution of MLTC clusters according to their specific burden indicators at each life decade for depression (cases) and their matched comparators (controls).** AUD=Alcohol use disorder; Valvular HD- Valvular Heart Disease; MSK=Musculoskeletal; CVD=Cardiovascular disorders; LowMLTC=low multiple long term conditions; CKD=Chronic kidney disease


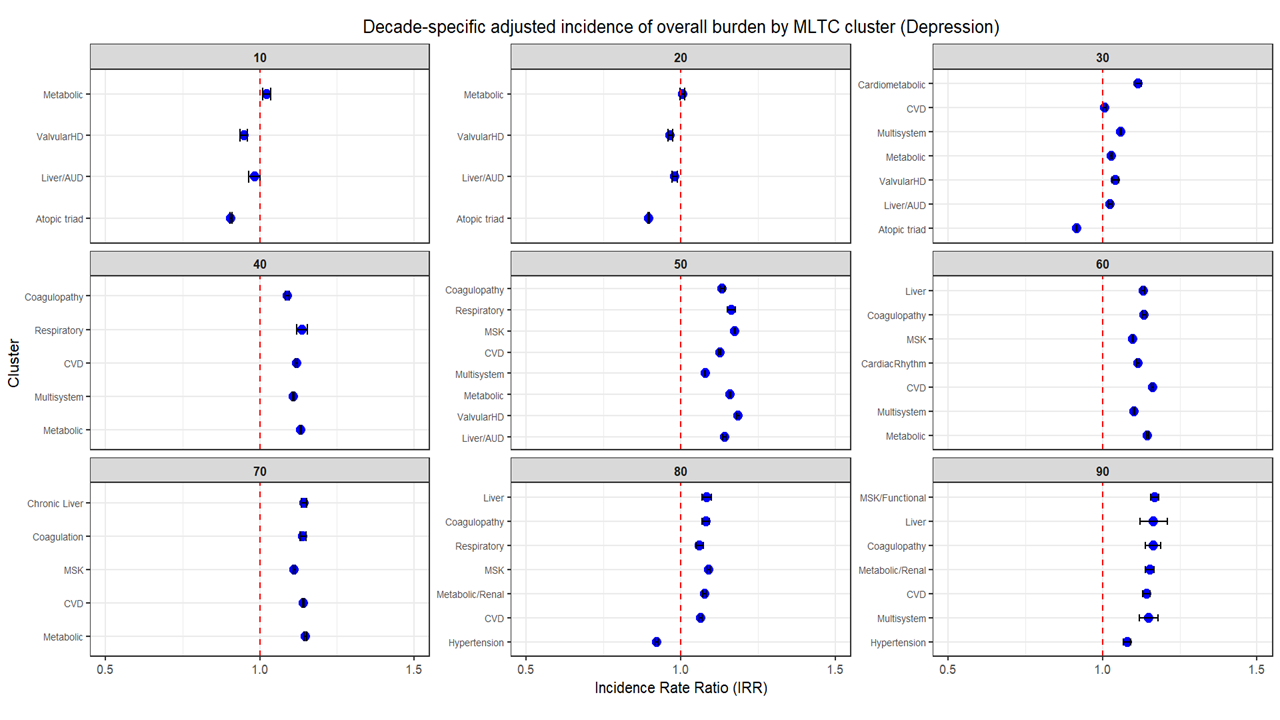


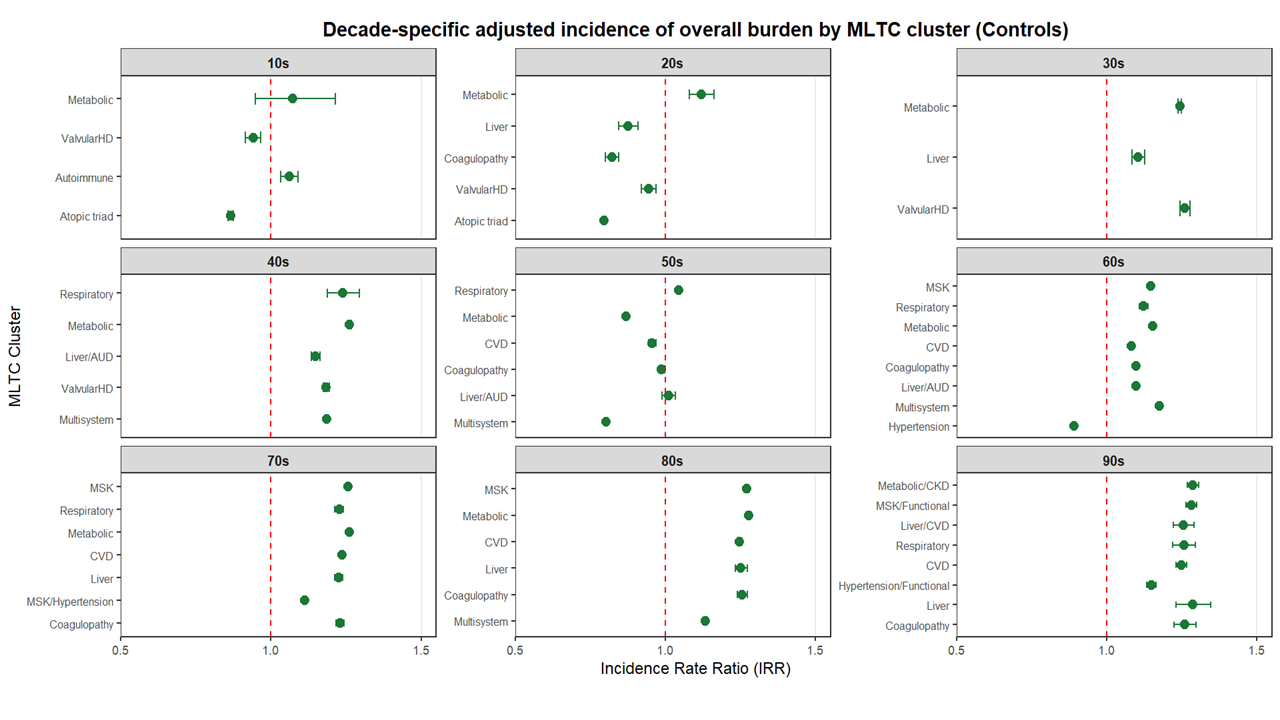


**Figure S5. The association between clusters of MLTCs with overall burden at each life decade for depression (top panel) and their comparators (bottom panel).** All associations are fully adjusted for age, sex, ethnicity, and region of registered GP practice and using robust clustering to recover standard errors. All reporting is with the LowMLTC cluster as the reference category for all decades. Associations are incidence rate ratio of elevated overall burden between having a MLTC in the cluster and not, such that an IRR of 1.15 would mean a mean count of burden is 15% higher than the reference category. Dots = IRR and bars represent 95% confidence intervals.


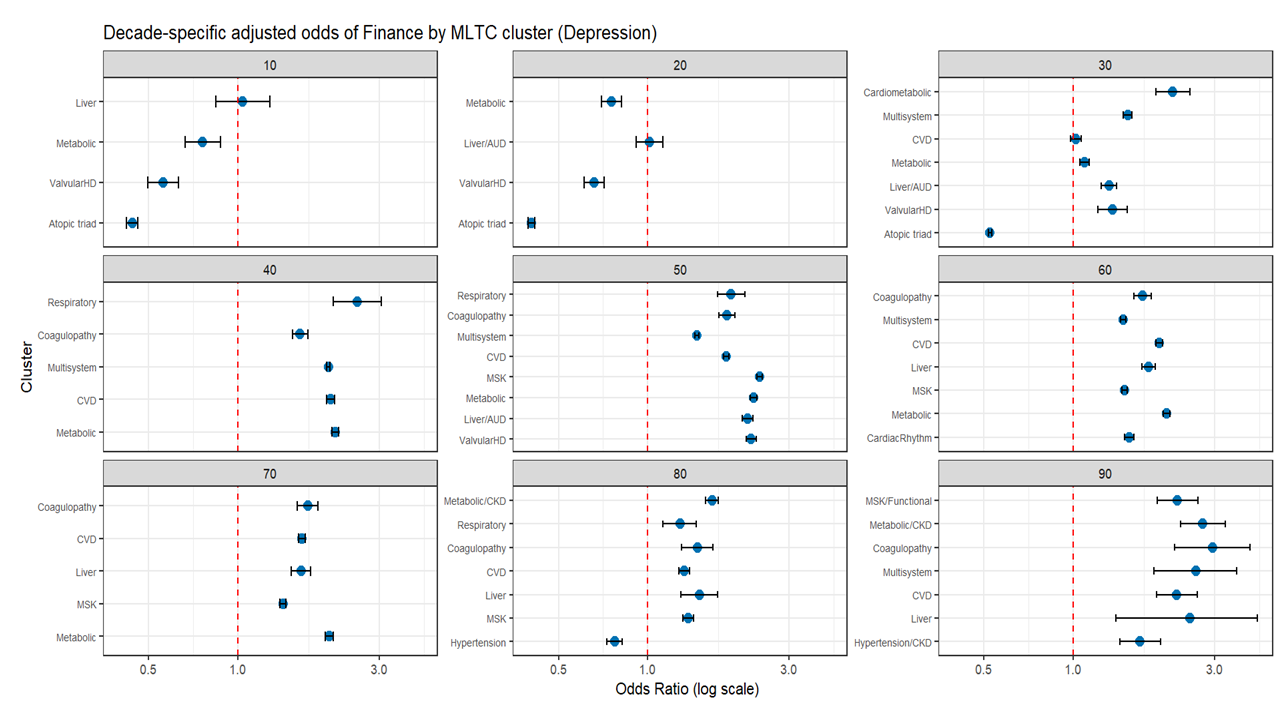


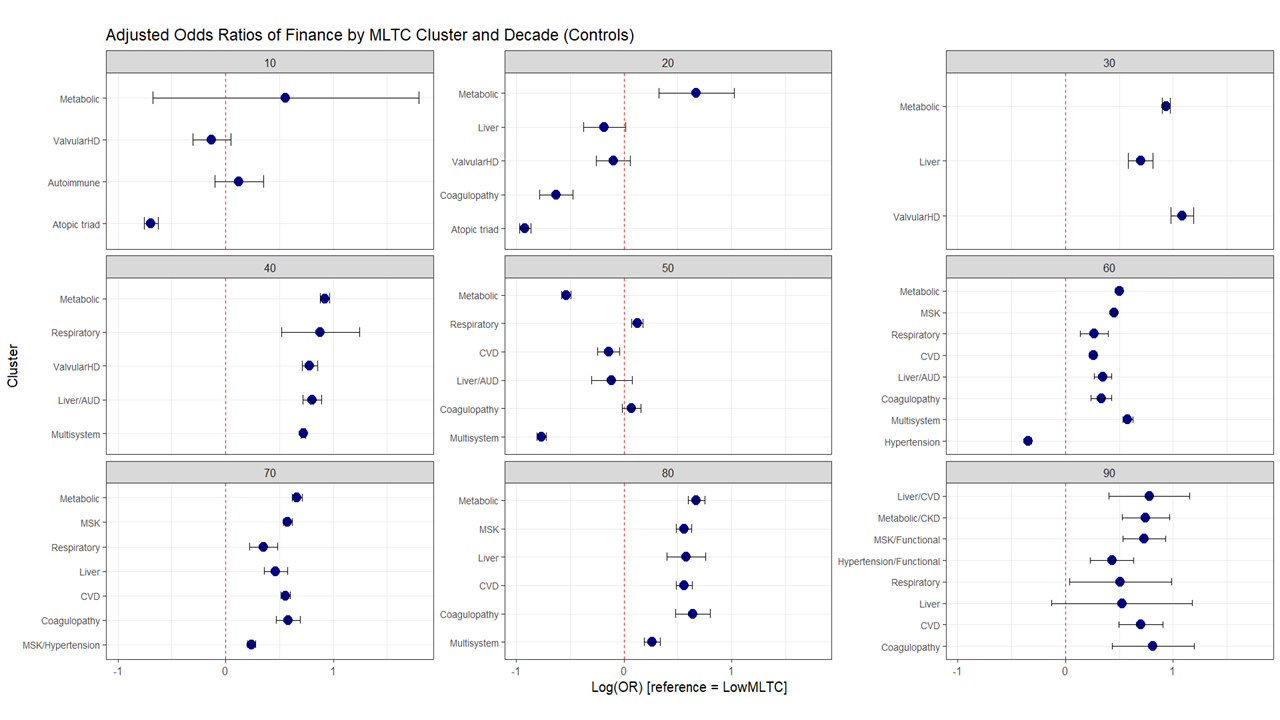


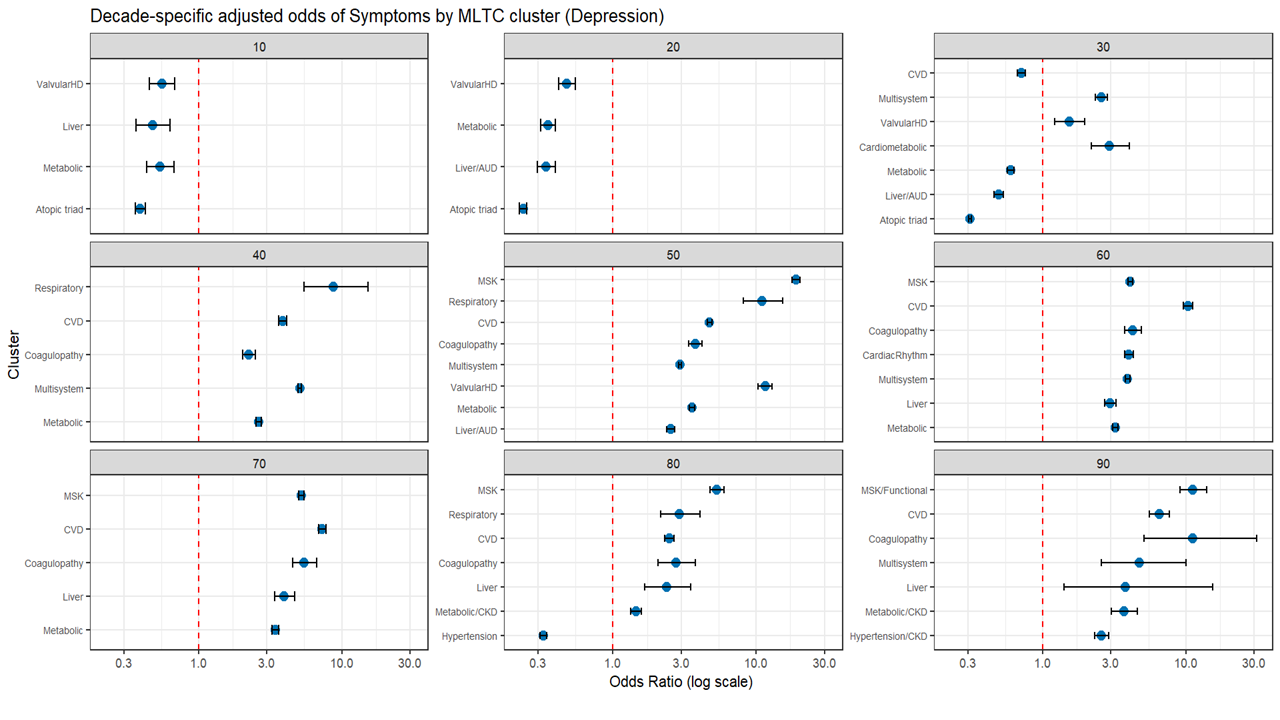


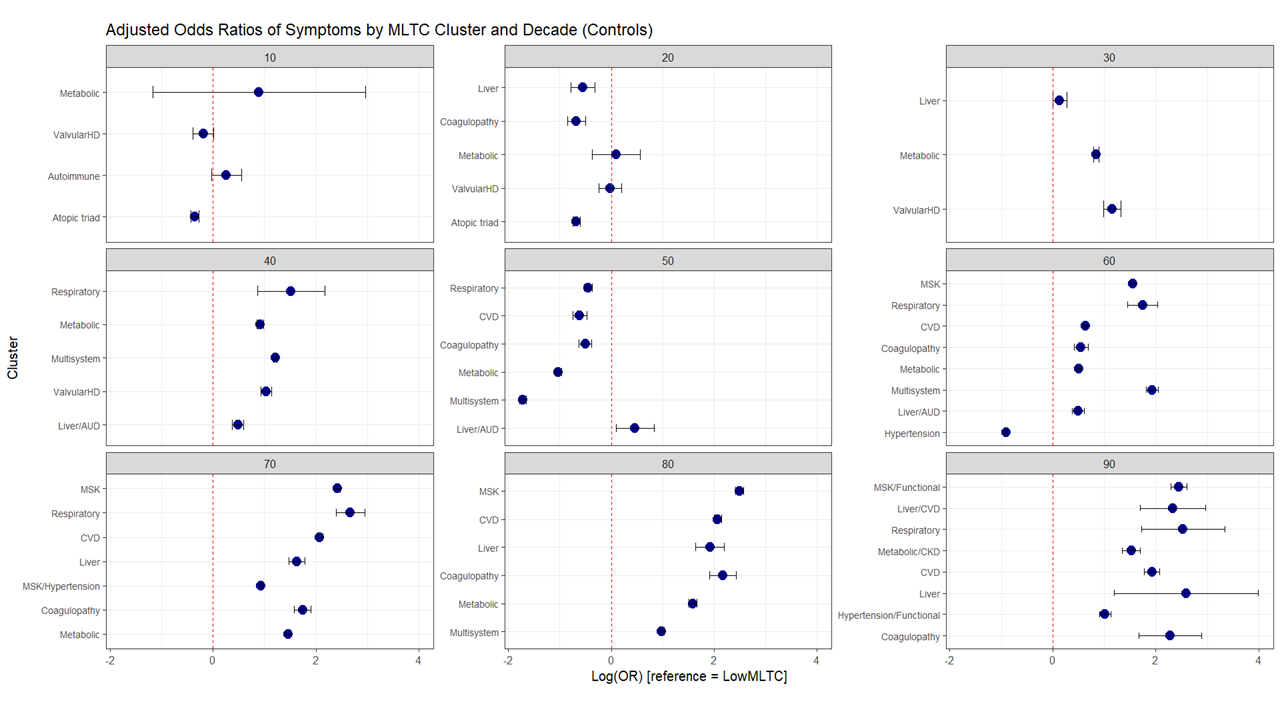


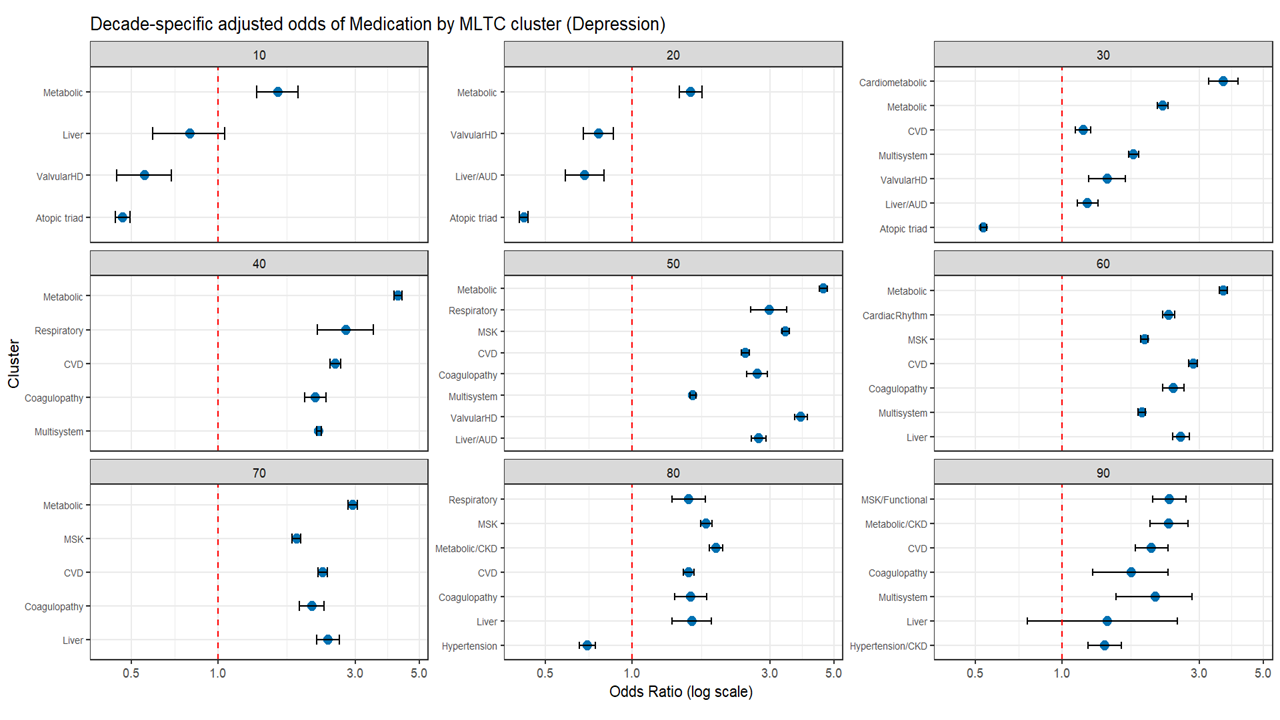


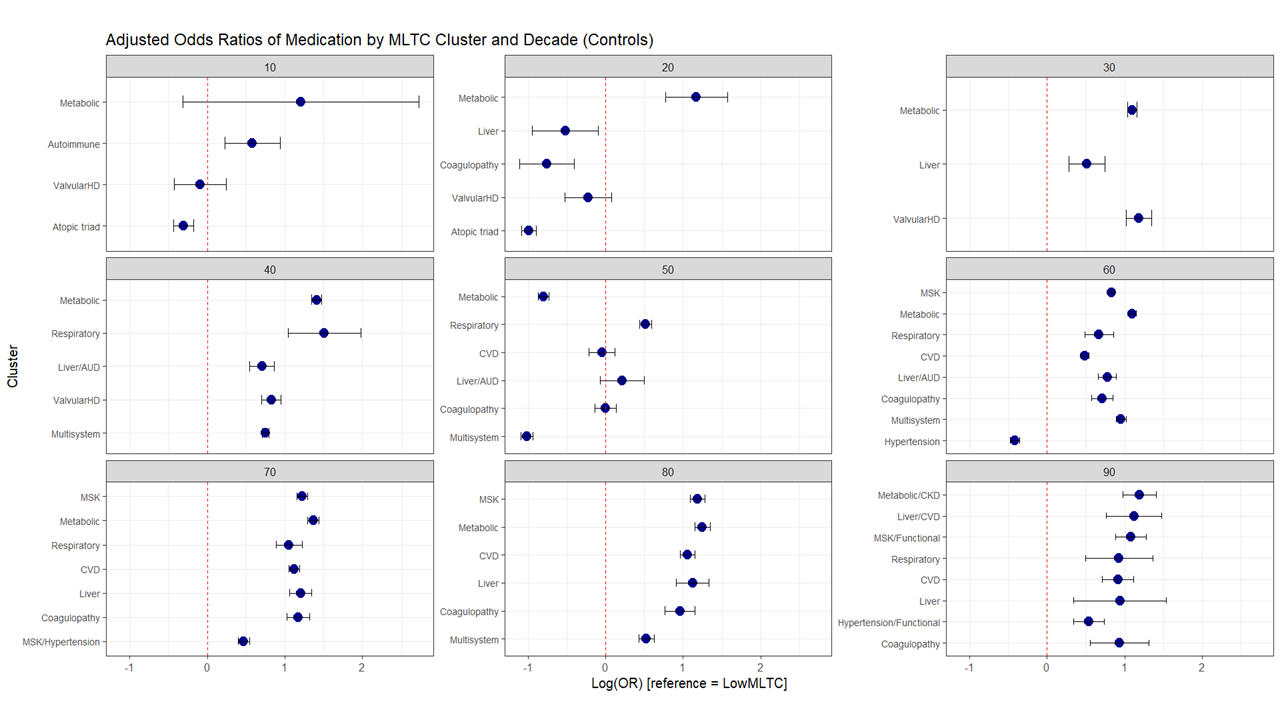


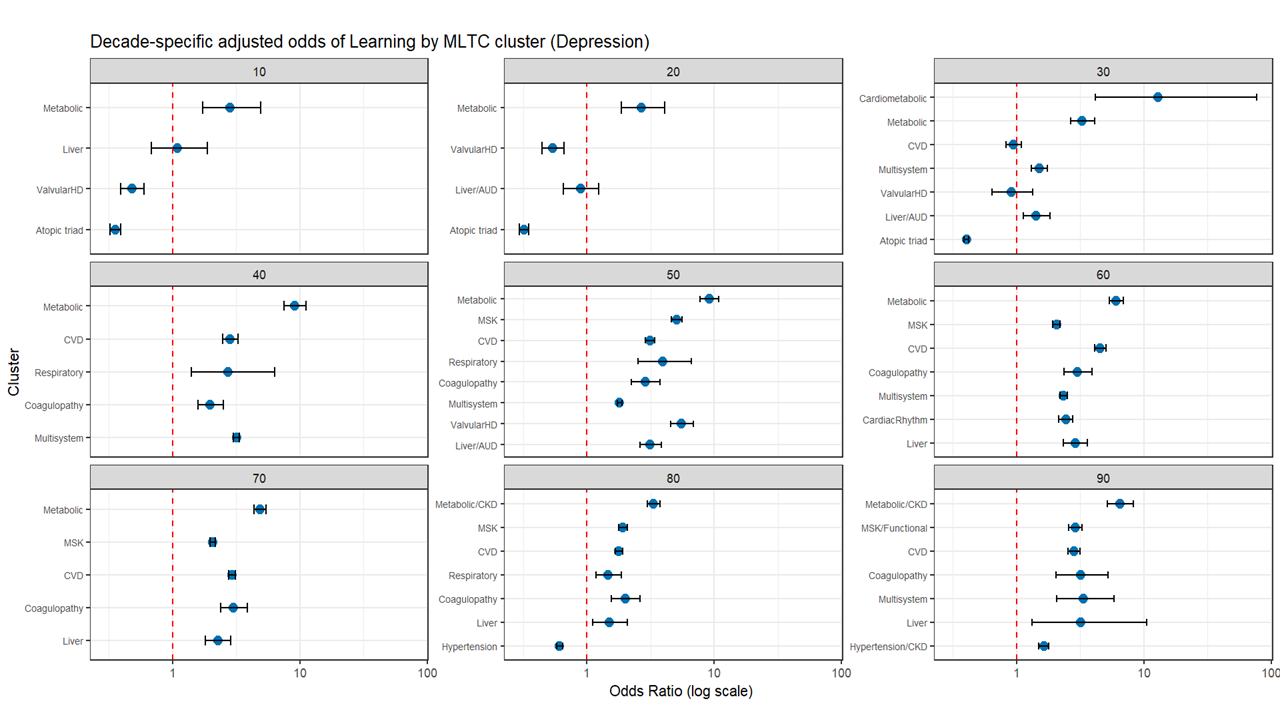


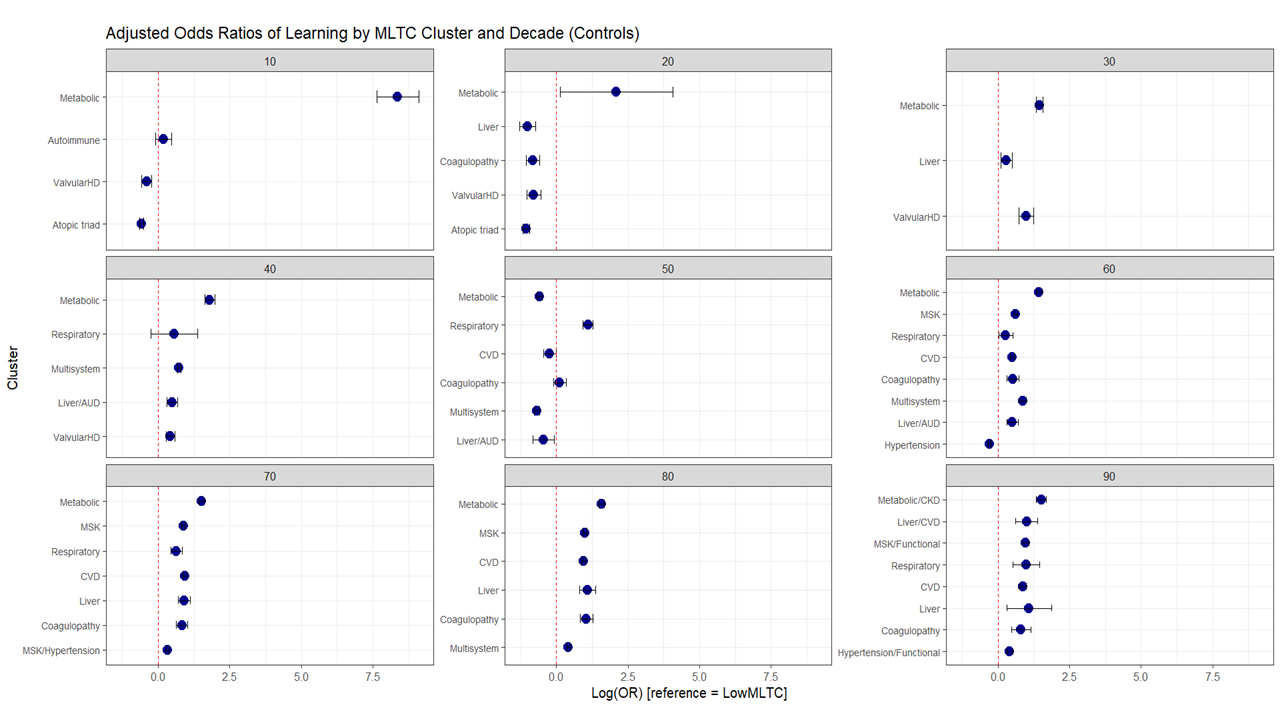


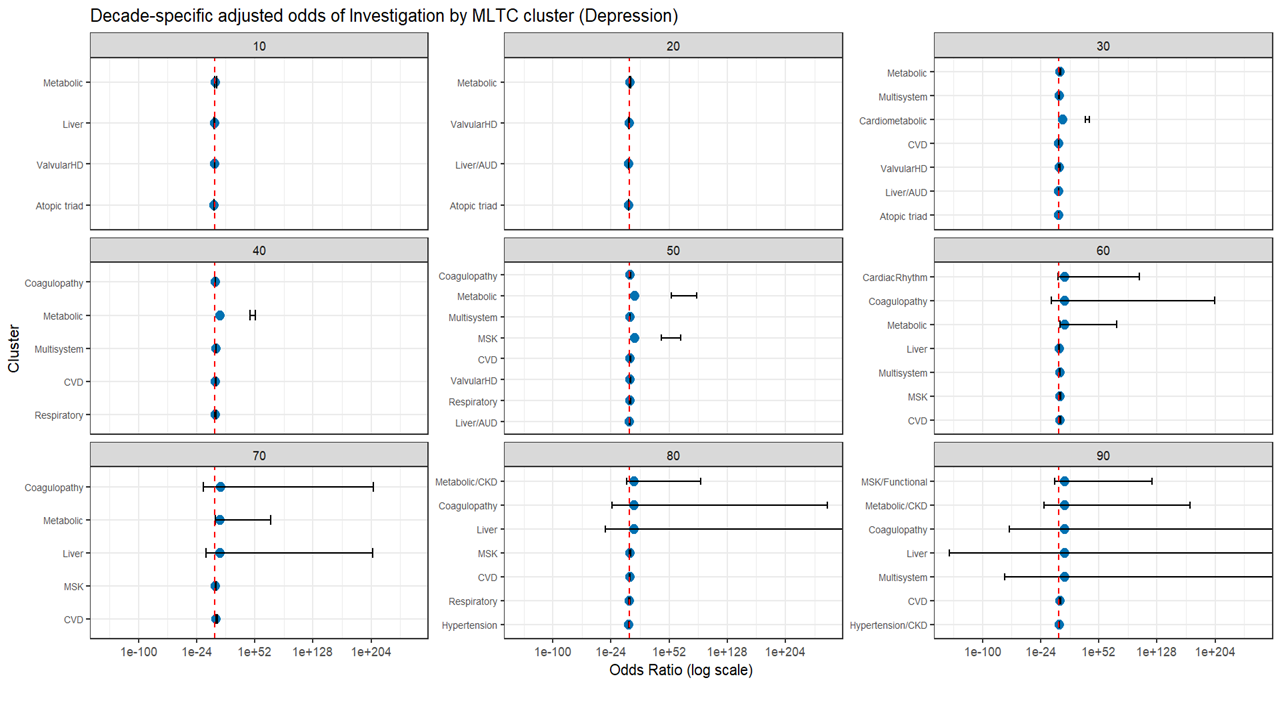


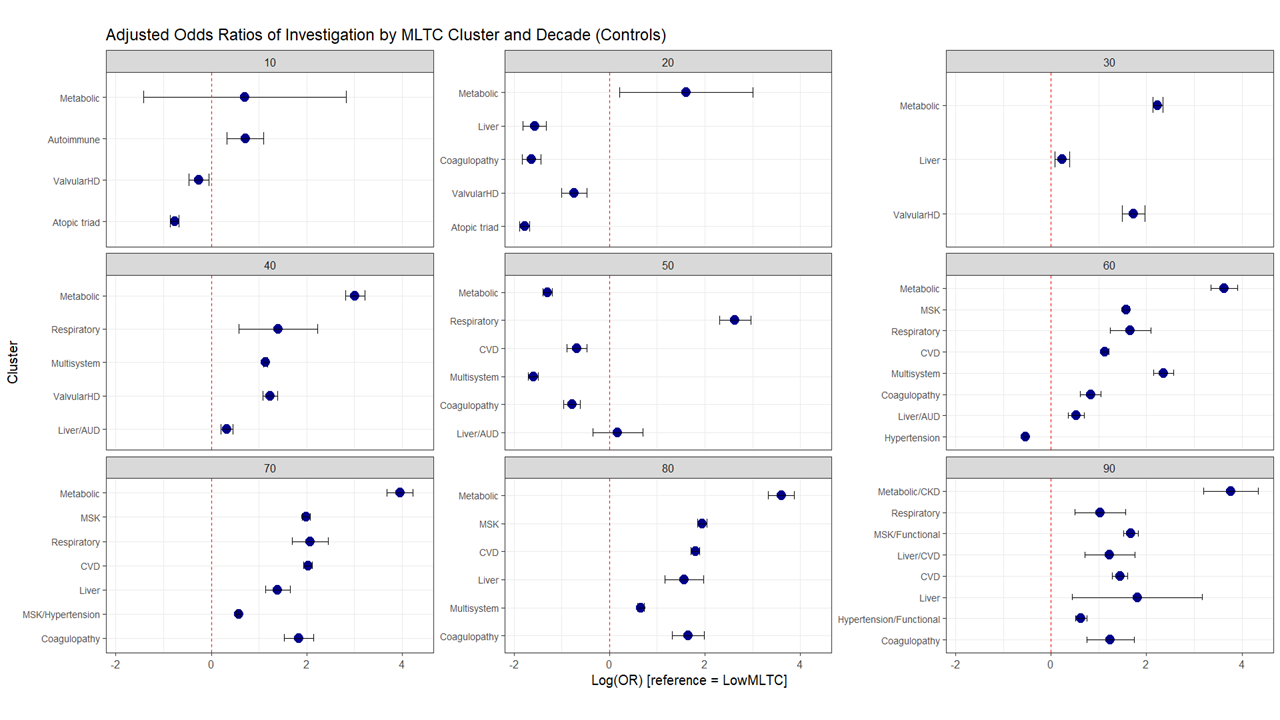


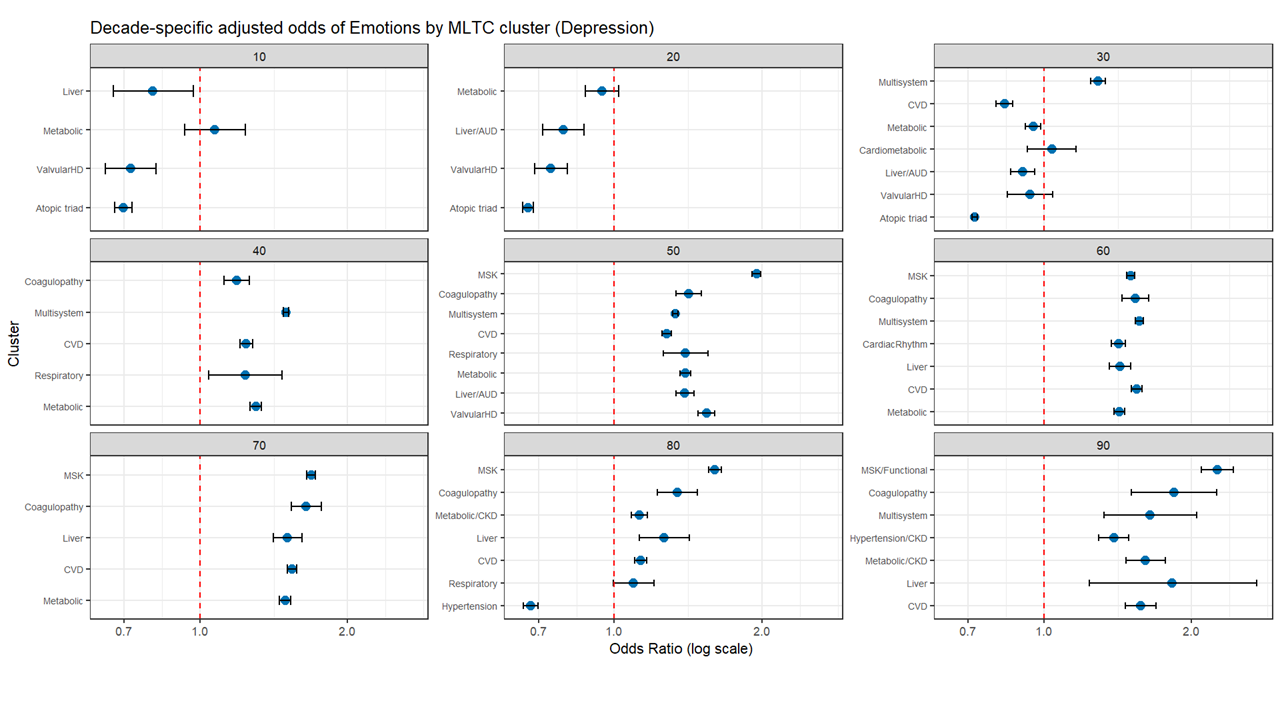


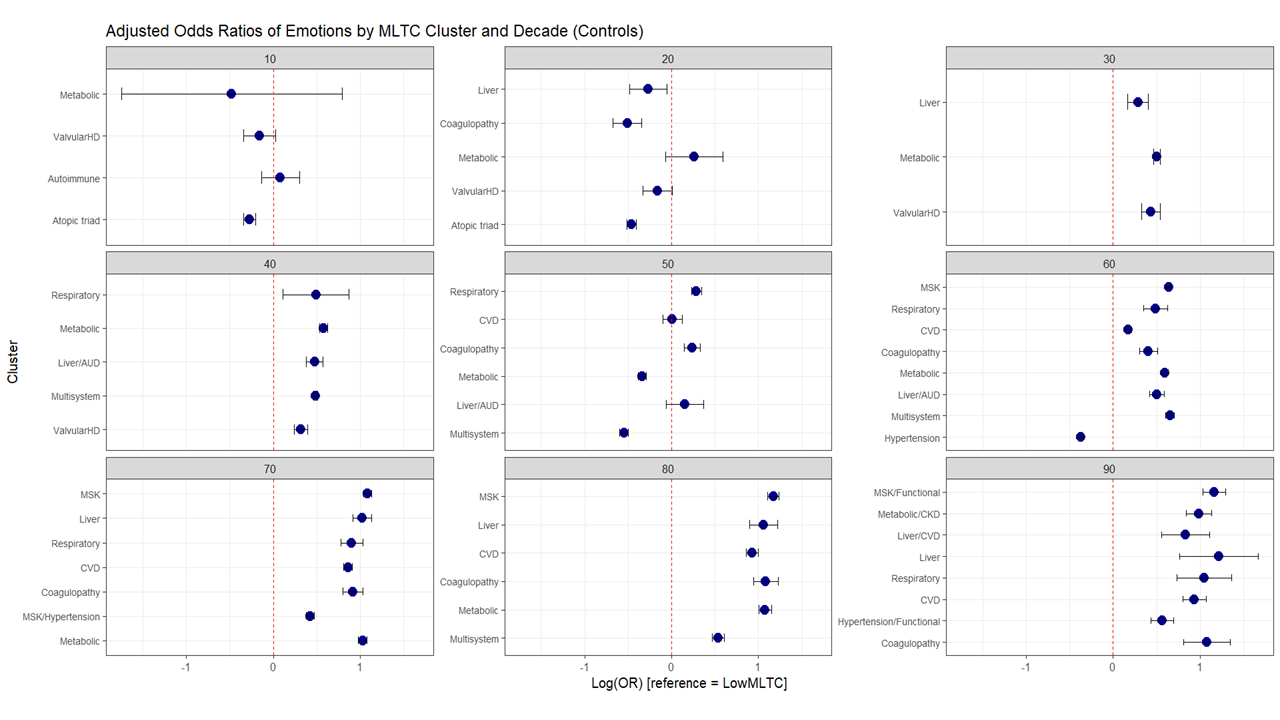


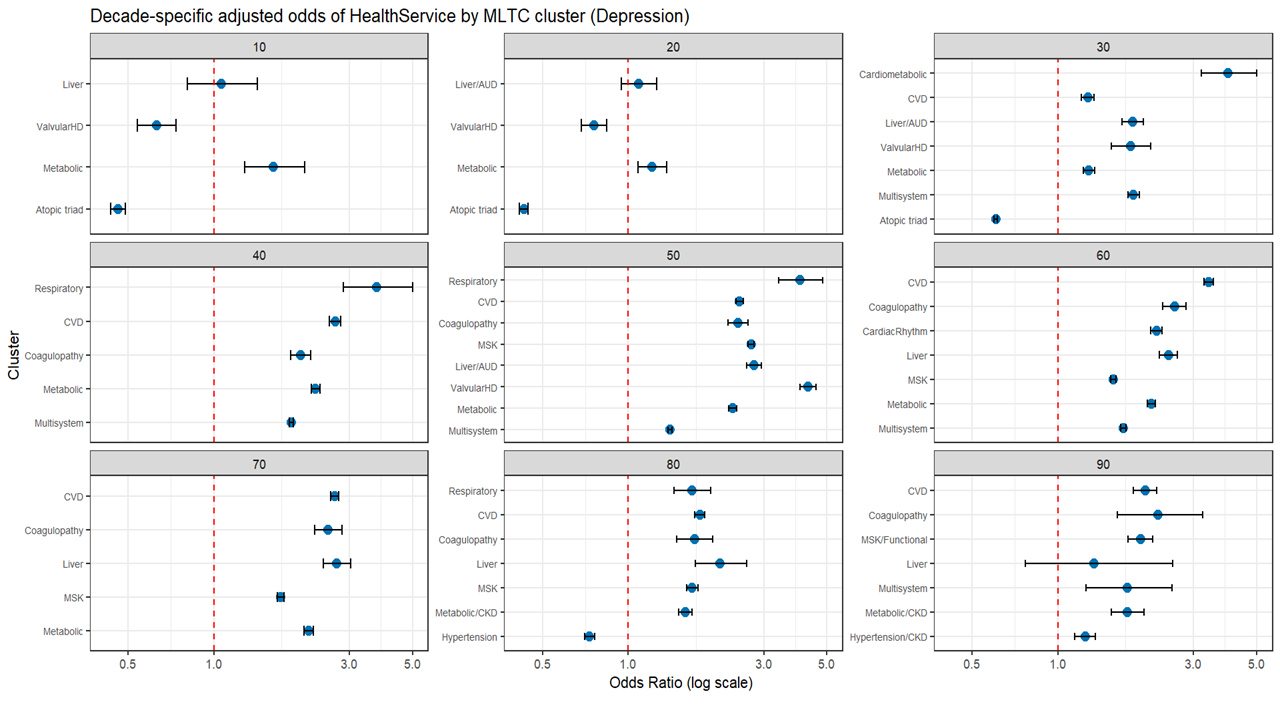


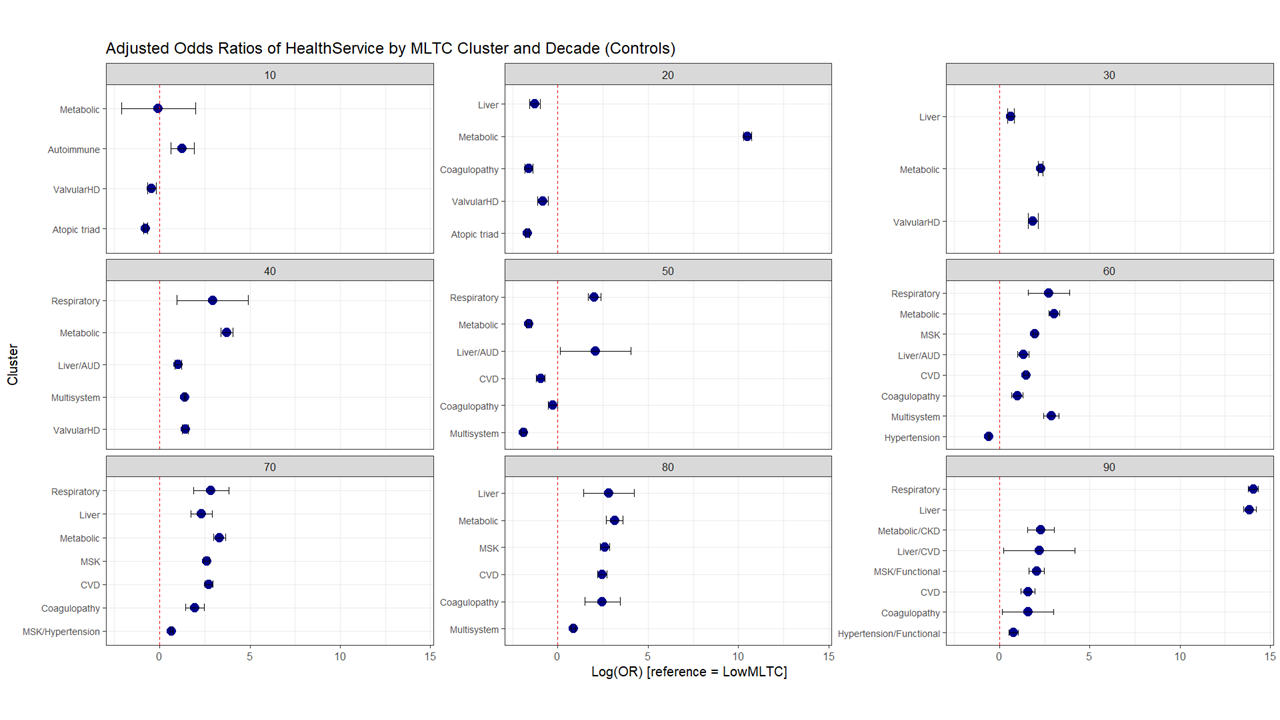


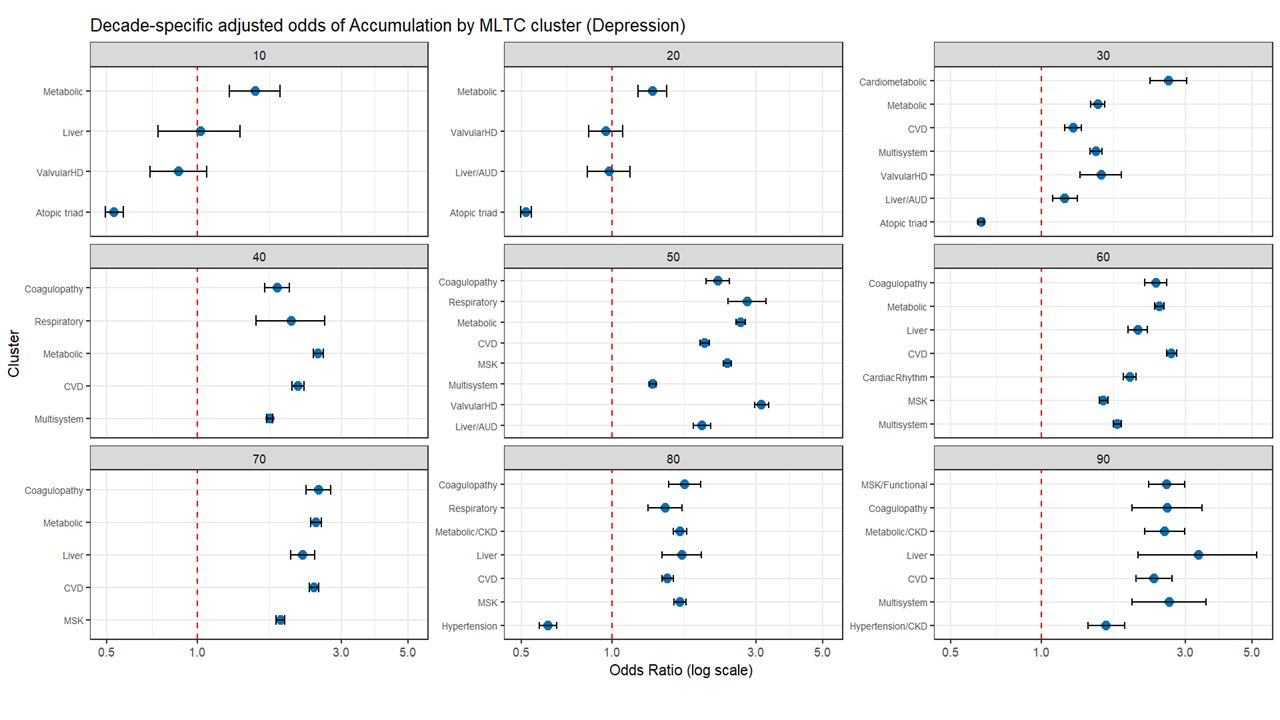


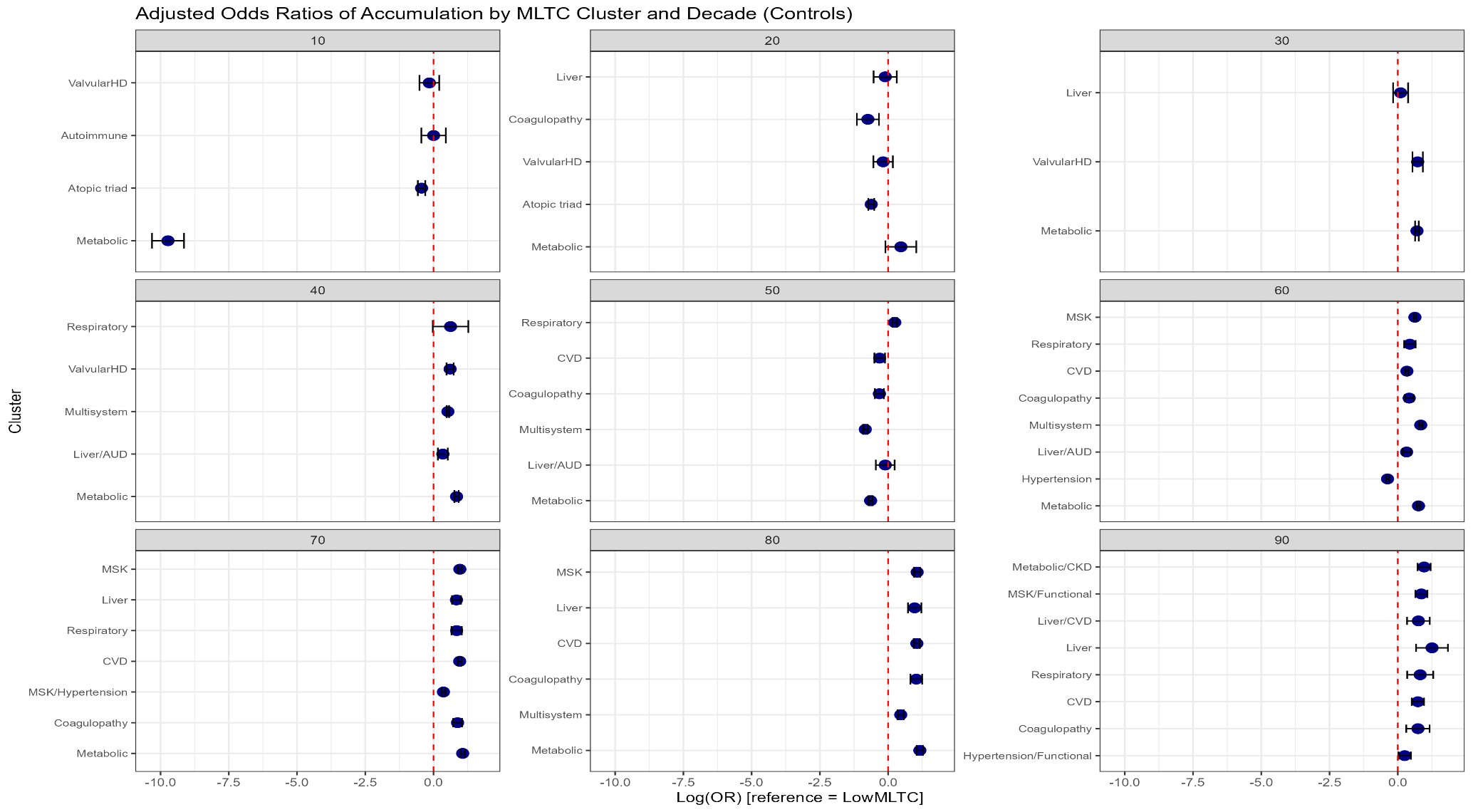


**Figure S6. Results of logistic regression analyses predicting individuals burden indicators at each life decade from decade-specific clusters of MLTC.** The analyses adjusted for age (year of birth), gender, ethnicity, and geographical region. AUD=Alcohol use disorder; Valvular HD- Valvular Heart Disease; MSK=Musculoskeletal; CVD=Cardiovascular disorders; LowMLTC=low multiple long term conditions; CKD=Chronic kidney disease


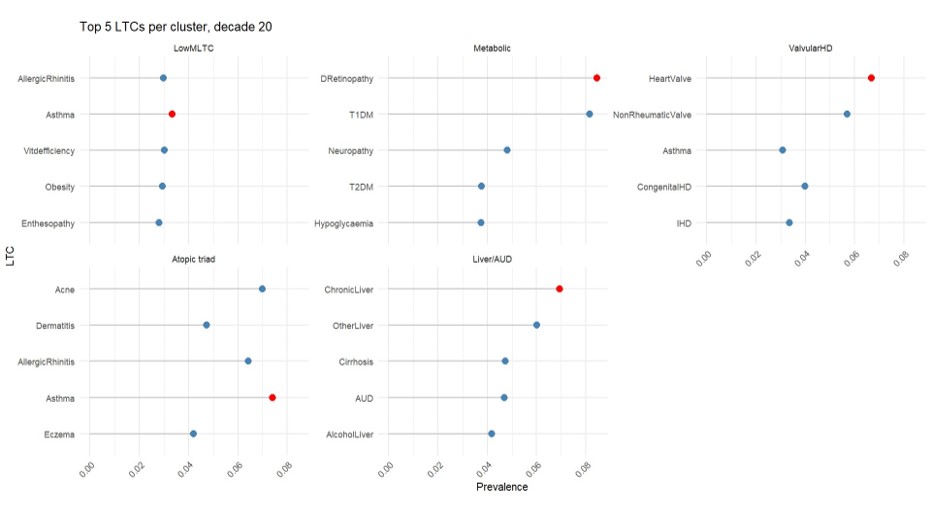


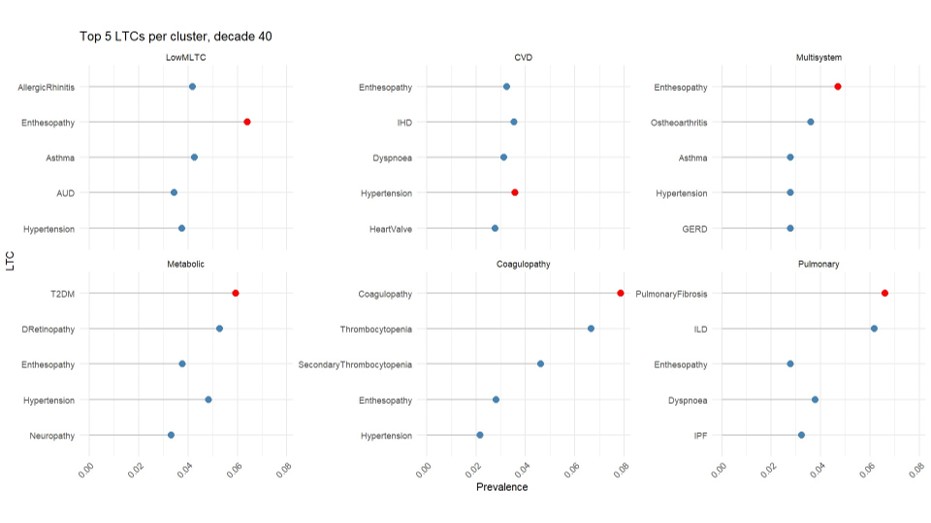


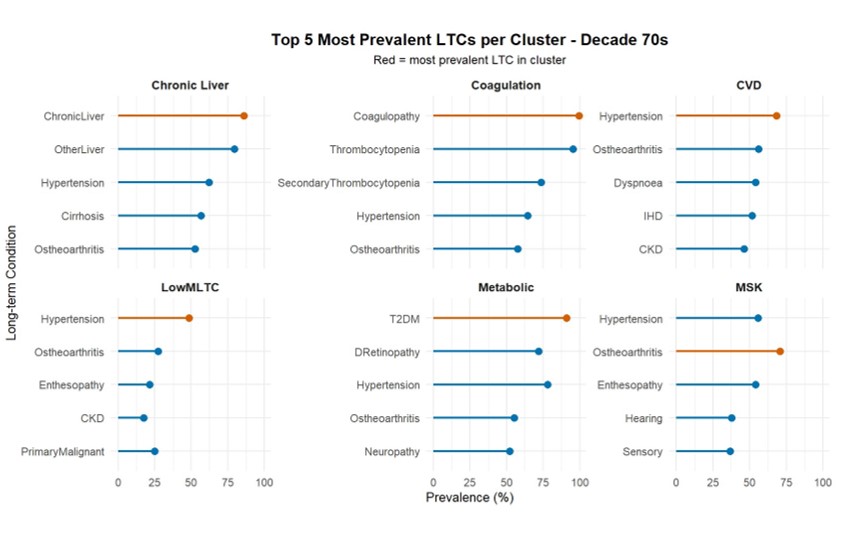


**Figure S7. Top 5 most prevalent LTCs per cluster for representative decades (20s- 40s- and 70s).**
